# Genetic Transformation of a Clinical (Genital Tract), Plasmid-Free Isolate of *Chlamydia trachomatis*: Engineering the Plasmid as a Cloning Vector

**DOI:** 10.1371/journal.pone.0059195

**Published:** 2013-03-18

**Authors:** Yibing Wang, Simona Kahane, Lesley T. Cutcliffe, Rachel J. Skilton, Paul R. Lambden, Kenneth Persson, Carina Bjartling, Ian N. Clarke

**Affiliations:** 1 Molecular Microbiology Group, University of Southampton, Southampton, United Kingdom; 2 Department of Virology, Ben Gurion University of the Negev, Beer Sheva, Israel; 3 Department of Laboratory Medicine, Malmo University Hospital, Malmo, Sweden; 4 Department of Obstetrics and Gynaecology, Malmo University Hospital, Malmo, Sweden; Louisiana State University Health Sciences Center, United States of America

## Abstract

Our study had three objectives: to extend the plasmid-based transformation protocol to a clinical isolate of *C. trachomatis* belonging to the trachoma biovar, to provide “proof of principle” that it is possible to “knock out” selected plasmid genes (retaining a replication competent plasmid) and to investigate the plasticity of the plasmid. A recently developed, plasmid-based transformation protocol for LGV isolates of *C. trachomatis* was modified and a plasmid-free, genital tract *C. trachomatis* isolate from Sweden (SWFP-) was genetically transformed. Transformation of this non-LGV *C. trachomatis* host required a centrifugation step, but the absence of the natural plasmid removed the need for plaque purification of transformants. Transformants expressed GFP, were penicillin resistant and iodine stain positive for accumulated glycogen. The transforming plasmid did not recombine with the host chromosome. A derivative of pGFP::SW2 carrying a deletion of the plasmid CDS5 gene was engineered. CDS5 encodes pgp3, a protein secreted from the inclusion into the cell cytoplasm. This plasmid (pCDS5KO) was used to transform *C. trachomatis* SWFP-, and established that pgp3 is dispensable for plasmid function. The work shows it is possible to selectively delete segments of the chlamydial plasmid, and this is the first step towards a detailed molecular dissection of the role of the plasmid. The 3.6 kb β-galactosidase cassette was inserted into the deletion site of CDS5 to produce plasmid placZ-CDS5KO. Transformants were penicillin resistant, expressed GFP and stained for glycogen. In addition, they expressed β-galactosidase showing that the lacZ cassette was functional in *C. trachomatis*. An assay was developed that allowed the visualisation of individual inclusions by X-gal staining. The ability to express active β-galactosidase within chlamydial inclusions is an important advance as it allows simple, rapid assays to measure directly chlamydial infectivity without the need for plaquing, fluorescence or antibody staining.

## Introduction

Members of the *Chlamydia* are obligate intracellular bacteria of significant importance in both human and animal pathogenesis. These microorganisms share a unique developmental cycle, in which a metabolically active reticulate body (RB) gives rise to a dense, infectious elementary body (EB) [1]. The process occurs within a specialised, chlamydia – modified cytoplasmic compartment known as an inclusion.

*Chlamydia trachomatis* can be divided into two major biological groups or biovars, those that cause Lymphogranuloma venereum (LGV biovar) which are highly infectious and cause an invasive sexually transmitted infection typically involving lymph nodes and can be spread systemically [2]. By contrast, the vast majority of *C. trachomatis* isolates are restricted to the mucosal epithelia of either the genital tract or the eye and comprise the ‘trachoma biovar’. *C. trachomatis* that belong to the trachoma biovar are the major infectious agents of preventable blindness in the developing world [3] and also the commonest cause of non-specific urethritis in developed countries [4]. Sexually transmitted infections caused by *C. trachomatis* (trachoma biovar) are frequently undiagnosed in women, leading to ascending infections that may progress to pelvic inflammatory disease, salpingitis and consequent infertility [5].

Recently we developed a transformation system for LGV isolates of *C. trachomatis* based on using the endogenous chlamydial plasmid pSW2 to generate a shuttle vector that replicates in both *E. coli* and *C. trachomatis* L2 [6]. The *E. coli* components of the shuttle vector were inserted within coding sequence 1 (CDS1) of the plasmid which had been inactivated by a naturally occurring deletion. In our previous study all transformations were only performed using laboratory-adapted LGV isolates that grow rapidly and have no requirement for centrifugation for cell infection. In the current study our initial aim was to extend the ‘proof of principle’ of *C. trachomatis* LGV transformation to a trachoma biovar isolate that is slower growing and has significantly lower infectivity than LGV isolates. It is important because these plasmid modified trachoma biovariants might also ultimately be more acceptable as attenuated delivery vehicles for a live *C. trachomatis* vaccine.

Nearly all strains of *C. trachomatis* carry an endogenous 7,500 bp plasmid; a few plasmid-free clinical isolates have been described although these are exceedingly rare [7]–[12]. Studies from plasmid-cured and naturally occurring plasmid-free *C. trachomatis* have shown that the presence of the plasmid is associated with the ability to accumulate glycogen, TL2 activation and infectivity [9], [13], [14]. *In vivo* studies using a murine model and plasmid deficient *C. muridarum* have shown that the plasmid is a virulence factor [15] although plasmid-free *C. caviae* do not show reduced virulence [16]. By contrast, experiments infecting the monkey eye with a plasmid–cured trachoma isolate of *C. trachomatis* have demonstrated that this isolate is avirulent and can also act as a vaccine [17].

There is thus a great deal of interest in understanding both the biochemical function and biological role of the plasmid especially since there are subtle differences between chlamydial species, our focus is on *C. trachomatis* [18]. Eight major coding sequences (CDS) (>100 bases) have been assigned to the chlamydial plasmid [19]–[23]. There are four 22-bp tandem repeats located in the intergenic region between CDS8 and CDS1, together with an upstream AT-rich region. These features are indicative of an origin of replication which has been confirmed by electron microscopy [24]. The positioning of CDS1 and CDS2 downstream of the tandem 22 bp repeated sequences, coupled with their size and net positive charge of their predicted products (sharing 32–35 % amino acid sequence identity), indicates that they are replication proteins [25]. The coding region of CDS1 is a naturally mutable part of the plasmid and there are numerous examples of this plasmid with premature stop codons and small deletions within CDS1. The most notable is the Swedish *C. trachomatis* new variant which carries a 377 bp deletion in CDS1 [26], [27]. CDS 3 encodes a protein with homology to *DnaB* from *E. coli*, this gene encodes a helicase enzyme involved in unwinding DNA during replication [21]. The proteins encoded by CDS 7 and 8 share features in common with plasmid partition proteins [25]. Thus CDS 2, 3, 7 and 8 are likely to be essential for plasmid replication.

Specific antibodies, raised to the predicted protein products encoded by each CDS have been used to investigate the expression profile for the 8 genes [28]. All 8 encoded proteins are detectable within the inclusion and their regulation is co-ordinated to the developmental cycle. Seven of the protein products of the CDSs are only associated with the inclusion and appeared to specifically stain EBs or RBs, consistent with a role in plasmid replication. Unusually, the protein ‘pgp3’ encoded by CDS5 is secreted beyond the inclusion and into the cell cytoplasm. This strongly suggests that the role of pgp3 is not related to the regulation of plasmid functions. Pgp3 assembles into a trimer and is membrane associated [29]. The mechanism by which this protein reaches the cell cytosol is unknown although it has been speculated that it might be an autotransporter rather than secreted through the type 3 mechanism. Antibodies to the assembled trimer of pgp3 are commonly produced in human chlamydial infection and are reported to be protective. Exogenous expression of pgp3 in the cytoplasm of *C. trachomatis* infected cells does not affect the *C. trachomatis* developmental cycle. Whilst there is high overall conservation (<1 % variation) of plasmid DNA sequences within *C. trachomatis* [19]–[22], [30], CDS5 appears to be the most variable region of the plasmid with the highest density of SNPs [27], [31]. Taken together these observations strongly suggested that pgp3 does not have a role in plasmid replication or maintenance. Thus our secondary aim was to test this hypothesis by creating a CDS5 knock out plasmid.

To check for essential function in our CDS5 ‘knock out’ plasmid vector we needed a *C. trachomatis* host that was plasmid-free. We obtained a naturally occurring plasmid-free genital tract *C. trachomatis* isolate from Sweden [32]. In this study we confirm that transformation of this little-passaged, clinical isolate from the genital tract as a recipient host is possible and that the plasmid gene encoding pgp3 is dispensable. Finally, we sought to investigate the plasticity of the chlamydial plasmid as a vector and to test whether it was possible to replace the gene encoding pgp3 with an unrelated expression/reporter cassette. For this purpose we selected the β-galactosidase operon as it has been extensively used to study gene expression in bacteria [33]. β-galactosidase is a large inducible multi-subunit enzyme encoded by a large operon (>3.5 kb) absent from the *C. trachomatis* genome [34]. We show that pgp3 can be replaced with this functional cassette and thus these studies open the way for developing new improved vectors that will lead to better *C. trachomatis* assays by direct staining of inclusions, an important first step in molecular dissection of plasmid functions.

## Materials and Methods

### Ethics Statement

All genetic manipulations and containment work was approved under the UK Health and Safety Executive Genetically Modified Organisms (contained use) regulations 2000 notification no GM57,10.1 entitled ‘Genetic transformation of Chlamydiae’.

### *C. trachomatis* Strain SWFP-

*C. trachomatis* SWFP- (Sweden F strain plasmid minus) is a plasmid-free serovar F strain isolated in Malmo, Sweden [32]. Two plasmid-free serovar F strains were isolated in 1995 from a couple (in their 20s) in a stable sexual relationship. The infection had been acquired abroad by one of them. The *C. trachomatis* used in this study was isolated in cell culture from a cervical swab. As expected, this isolate requires centrifugation to initiate cell infection and displays the typical *C. trachomatis* developmental cycle and compact inclusions in McCoy cells at 48 hrs post infection.

### Propagation of *C. trachomatis* Strain SWFP-

McCoy cells were used for propagation of *C. trachomatis* SWFP- and for the transformation studies. They were grown in Dulbecco’s modified Eagles’ medium (DMEM) supplemented with 10% fetal calf serum (FCS). Cells were incubated with *C. trachomatis* SWFP- inoculum and centrifuged with a Beckman Coulter Allegra X-15R Benchtop Centrifuge at 1800 rpm (or 754×g) for 30 min to initiate cell infection. Then the culture medium was replaced with fresh medium containing cycloheximide (1 µg/ml) and gentamicin (25 µg/ml). Two days after infection, the cells were harvested using cell scrapers, and then spun at 3500 rpm or 2851×g for 10 min. The cell pellet was saved, resuspended in 1 ml of cold 10% PBS, and transferred into a bijoux tube with glass beads. The cells were lysed by vortexing for 1 min. The cell debris was removed by spinning at 1000 rpm or 233×g for 5 min. The supernatant was saved (∼ 1 ml) and mixed with an equal volume of phosphate buffer containing 0.4 M sucrose (4SP), and stored at −80°C.

### Purification of Chlamydial EBs and RBs

*C. trachomatis* EBs and RBs were purified using two cycles of density gradient centrifugation as previously described [35]. Samples harvested from infected cells were overlayed on a 20% Urografin (Schering Healthcare, UK) in PBS and centrifuged at 100 000×*g* for 2 h in a Beckman SW28 rotor. The pellet was collected and resuspended in 2 mL of PBS. The chlamydia were further purified by overlaying onto a discontinuous urografin gradient that consisted of 34, 44 and 54% urografin in PBS and centrifuging at 100 000×*g* for 2 h in a Beckman SW28 rotor. RBs banded at the 34%/44% interface and EBs banded at the 44%/54% interface. The RB and EB bands were collected and were pelleted by centrifugation at 35 000×*g* for 30 min in a Beckman 55.2 rotor. The pellets were resuspended in PBS and stored in aliquots at −80°C.

### Recombinant Plasmid Vectors for Transformation

#### 1. Plasmid pGFP::SW2

The construction of pGFP::SW2 was described previously [6]. It contained the pSP73 backbone with pSW2 (from *C. trachomatis* SW2) inserted at the unique BamHI site and a fused *gfpcat* gene (under the control of a promoter derived from *Neisseria meningitidis*) at PstI/SalI sites.

#### 2. Construction of the knockout vector pCDS5KO

Plasmid pCDS5KO is based on pGFP::SW2. The only difference is that the 655 bp PacI-BsaBI fragment in pGFP::SW2 was replaced with a 35 bp oligo 5′-TAATAGCAAGCTTGAAACTAAAAACCAGGCCTGAT-3′, so that the promoter of CDS5 and ¾ of CDS5 coding region were deleted; a unique StuI site was included as an additional cloning site. pCDS5KO was constructed by replacing the 1422 bp EcoRI (in CDS4) and BsaBI (in CDS5) fragment from pGFP::SW2 with a 802 bp EcoRI-EcoRV fragment from CDS4 PCR product using DNA template pGFP::SW2 and primers ORF4_F(RI) (5′-AAAACTGAATTCTTAGAGGCTTATGG-3′) and ORF4_R(RV) (5′-AAAAAAGATATCAGGCCTGGTTTTTAGTTTCAAGCTTGCTATTA-3′). The clone was made by ‘three fragment ligation’: two restriction endonuclease cleavage fragments from pGFP::SW2 (8101 bp BsaBI fragment and 2016 bp BsaBI-EcoRI fragment) and a 802 bp EcoRI-EcoRV fragment from the CDS4 PCR product, resulting in a 629 bp deletion from pGFP::SW2 (between the 24^th^ bp after the stop code of CDS4 and the BsaBI site on CDS5). The PCR product and ligations sites, including the intact CDS4 were sequence verified. The nucleotide sequence and features of pCDS5KO are shown in Figure S1.

#### 3. Construction of the vector with *lacZ* (placZ-CDS5KO)

Plasmid placZ-CDS5KO was constructed by cloning a modified *lacZ* cassette into the unique StuI site in pCDS5KO. The *lacZ* cassette contains a ∼200 bp promoter from chlamydiaphage Chp2 ORF5 (Chp2P5) in tandem with the minimal gpt promoter for *lacZ* from pSV-B-Gal (Promega). placZ-CDS5KO was constructed in two steps: firstly, the 226 bp BamHI-HindIII fragment of Chp2P5 and the 3749 bp HindIII-SalI fragment of *lacZ* from pSV-B-Gal (Promega) were cloned into the BamHI and XhoI sites of pSP73 to get an intermediate plasmid pSP73-Chp2P5-lacZ. Then the 3855 bp Smal-PsiI fragment from pSP73-Chp2P5-lacZ (the *lacZ* cassette) was inserted into the unique StuI site of pCDS5KO. The nucleotide sequence and features of placZ-CDS5KO are shown in Figure S2.

### Genetic Transformation of *C. trachomatis* SWFP-

Transformation of *C. trachomatis* SWFP- was performed using the Calcium Chloride based transformation protocol developed for *C. trachomatis* L2 [6] with the following modifications: (1) centrifugation (1800 rpm or 754×g for 30 min) to initiate cell infection; (2) an increased amount of *C. trachomatis* SWFP- EB for the initial CaCl_2_ treatment; (3) selection of transformants in a T_25_ flask at passage 1. The experimental procedures were as follows: 1×10^8^ IFU of SWFP- EBs (concentrated to 10 µl) and 6 µg plasmid DNA (10 µl) were mixed in a total volume of 250 µl CaCl_2_ buffer (10 mM Tris pH 7.4 and 50 mM CaCl_2_) and then incubated for 30 min at room temperature. Freshly trypsinised McCoy cells (7×10^6^), resuspended in 250 µl CaCl_2_ buffer were then added to the plasmid/EB mix and incubated for a further 20 min at room temperature with occasional mixing. This mixture was aliquoted to six wells in a six-well plate with 2 mls/well of pre-warmed DMEM +10% FCS (without cycloheximide or penicillin). The cells were allowed to settle and incubated at 37°C in 5% CO_2_ for 1 hour before centrifugation at 1800 rpm or 754×g for 30 min. The plate was returned to the incubator for 2 days. Infected cells were scraped off from wells with 1 ml filter tip and bulk harvested (as described earlier) from six wells in 2 ml. This sample was called T_0_. The selection of transformants was performed by infection of McCoy cells in 2×T_25_ flasks using 0.5 ml of T_0_ (passage 1) and selected with 10 units/ml of penicillin. Two days after infection, the sample was harvested from 2×T_25_ flasks as ‘T_1_’. All T_1_ was used to infect McCoy cells in a T_25_ flask with 10 units/ml penicillin. Passaging was continued in T_25_ flasks with 10 units/ml of penicillin until normal inclusions were recovered. The transformants were routinely recovered in passages 2 or 3 (see Figure S3).

### Microscopy

Cells in culture and cells infected with *C. trachomatis* were routinely visualized by phase contrast microscopy using a Nikon eclipse TS100 inverted microscope with fluorescence accessories and a Nikon blue excitation fluorescence filter. Live phase contrast and fluorescence images were captured using a Nikon DS-Fi1 camera head fitted to the inverted microscope. *C. trachomatis* inclusions were stained with iodine as previously described [6]. Counting of inclusion forming units (IFU) to quantify chlamydial infectivity was performed on serial dilutions of *C. trachomatis* in monolayers of McCoy cells grown in 96 well trays. For this assay inclusions were immunostained as previously described [36].

### Time Course of Infection

McCoy cells grown to confluence in 24 well plates were infected with *C. trachomatis* at MOI = 1. For *C. trachomatis* L2(25667R) and *C. trachomatis* L2(25667R) transformed with plasmid pGFP::SW2, EBs were allowed to adsorb to cells for 1 h at 37°C; for *C. trachomatis* SWFP- and *C. trachomatis* SWFP- transformed with plasmid pGFP::SW2, the 24 well plates were spun at 1800 rpm or 754×g for 30 min at RT. The cells were then overlaid with fresh culture medium containing cycloheximide (1 µg/ml) and gentamicin (25 µg/ml), and incubated at 37°C in 5% CO_2_. The infection was stopped at 8-hourly time points (8, 16, 24, 32, 40 and 48 h) by scraping up the cells and vortexing with glass beads. The samples were then rapidly frozen and stored at −80°C and assayed for infectivity as previously described [6].

### Iodine Staining of Inclusions

Iodine staining of inclusions was previously described [6]. Briefly, *C. trachomatis* strains were cultured in McCoy cells on coverslips, and infected cells bearing *C. trachomatis* inclusions were washed with PBS and then fixed to coverslips with ice-cold methanol. The coverslips were stained with 5% iodine stain (containing both potassium iodide and iodine in 50% ethanol) for 10 min. The stain was then changed for 2.5% iodine stain for 10 min and mounted in 5% iodine stain in glycerol (1∶1) for photomicroscopy.

### Assay for β-galactosidase Expression in *C. trachomatis* Inclusions by X-gal Staining

McCoy cells were infected with *C. trachomatis* transformants SWFP−/pCDS5KO and SWFP-placZ-CDS5KO in 6-well or 24-well plates. Two days after infection, the cells were washed with 1×PBS and fixed in Formaldehyde fixative (1∶10 dilution of 37–41% Formaldehyde stock in 1×PBS) for 15 min. The cells were washed with 1×PBS before adding freshly prepared X-gal staining solution (1 mg/ml X-gal (from 40 mg/ml x-gal stock in DMSO), 2 mM MgCl2, 5 mM potassium ferrocyanide and 5 mM potassium ferricyanide in 1×PBS). The plates were covered with foil and put in a 37°C incubator overnight.

### Southern Blotting

Southern hybridization analysis was previously described [6]. Briefly, the DNA blot was probed with nonradioactive, digoxigenin-11-dUTP-labeled probes (Random primed DNA labelling) and chemiluminescence detection with CSPD (Roche Diagnostics Ltd., Product No. 11 585 614 910). The *gfp* probe was a 739 bp *Bam* HI/*Bgl* II fragment from pGFP::SW2, which annealed to a 1445 bp fragment of *Bam* HI digested pGFP::SW2 or a 3625 bp fragment of *Bgl* II digested pGFP::SW2.

#### Real-time QPCR

Our quantitative real-time PCR protocol to determine the absolute number of chlamydial plasmids and genomes in samples using 5′- exonuclease (TaqMan) assays with unlabelled primers and carboxyfluorescein/carboxytetramethylrhodamine (FAM/TAMRA) dual-labeled probes has been described previously [37]. Real-time PCR cycles were performed in an ABI PRISM 7700 Sequence Detection System (Applied Biosystems) according to the manufacturer’s instructions. Reactions were assembled using duplicate standard samples.

## Results

### Transformation of a Plasmid-free Genital Tract Isolate of *C. trachomatis*

**Figure 1:**
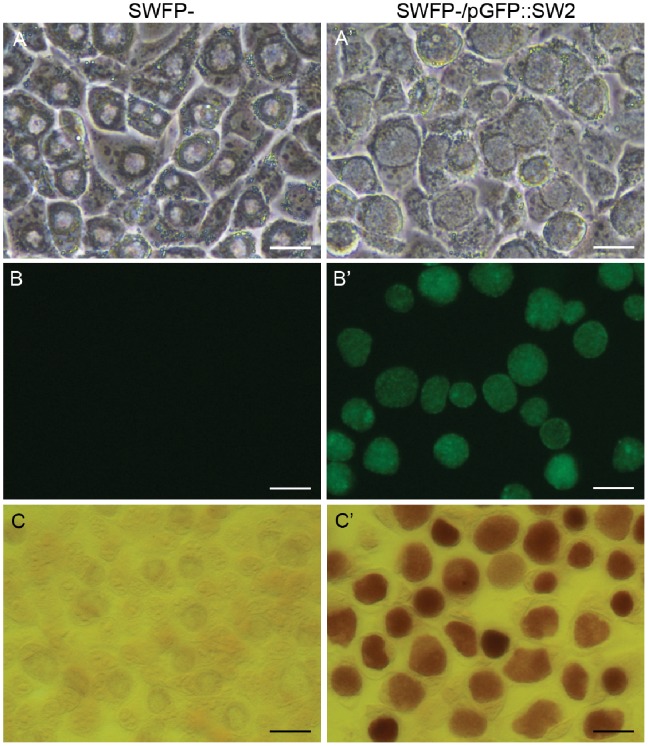
Properties of *C. trachomatis* SWFP^−^ and *C. trachomatis* SWFP^−^ transformed with plasmid pGFP::SW2 (SWFP^−/^pGFP::SW2). A to B’: Images of live McCoy cells infected with SWFP^−^ or SWFP^−/^pGFP::SW2 under white light (phase contrast, A and A’) and the same fields under blue light (B and B’). C and C’: Iodine stained McCoy cells (Methanol-fixed on coverslips) infected with SWFP^−^ (C) and SWFP^−/^pGFP::SW2 (C’). Mature SWFP^-^ and SWFP^−/^pGFP::SW2 inclusions are morphologically distinct by phase contrast microscopy (A and A’). The scale bar represents 20 µm.

Our initial aim was to transform a genital tract isolate of *C. trachomatis*; for this purpose we applied the original protocol for transformation of *C. trachomatis* L2 to the non-invasive genital tract (trachoma biovar), plasmid-free Swedish isolate *C. trachomatis* SWFP-. However, the infectivity of this plasmid-free isolate dropped dramatically following the CaCl_2_ treatment used to make these bacteria competent. Therefore the protocol was modified by increasing of the number of EBs and introducing a low speed centrifugation step which was necessary for efficient cell infection. By using this modified transformation protocol, transformants of *C. trachomatis* SWFP- with pGFP::SW2 were routinely recovered after 2–3 rounds of penicillin selection (Figure S3). Transformants of *C. trachomatis* SWFP- with the vector pGFP::SW2 displayed the typical *C. trachomatis* development cycle and compact inclusions characteristic of genital tract strains in McCoy cells at 48 hrs post infection (Figure 1A’). Expression of GFP was detected within each inclusion under blue light (Figure 1B’). Furthermore, inclusions of *C. trachomatis* SWFP- transformed with plasmid pGFP::SW2 had acquired the ability to accumulate glycogen as determined by iodine staining of inclusions (Figure 1C’).

Mature inclusions of the plasmid-free *C. trachomatis* SWFP- isolate have the characteristic ‘bulls-eye’ appearance previously described for other plasmid-free *Chlamydia* [14]. In these inclusions, the Brownian motion or pedesis is confined to the periphery, leaving a ‘hole-like’ space in the centre. However, transformation with the vector pGFP::SW2 restored the normal inclusion phenotype (Figure 1A’). A Southern blot was performed to ensure that the new phenotypes displayed by *C. trachomatis* SWFP- were related to the acquisition of pGFP::SW2 solely as an extrachromosomal element and were not due to its integration with the bacterial chromosome (potentially disrupting other genes). The Southern blot showed the presence of pGFP::SWP only as a supercoiled plasmid (Figure S4).

The pGFP::SW2-transformed *C. trachomatis* SWFP- appeared to grow at a faster rate than the untransformed *C. trachomatis* SWFP- parent. We also observed a similar, fast growth phenotype between the pGFP::SW2-transformed plasmid-free LGV isolate *C. trachomatis* L2(25667R) and the untransformed *C. trachomatis* L2(25667R) parent. To investigate this observation and place it within a quantitative framework, one-step growth curves were performed on the pGFP::SW2 transformed *C. trachomatis* SWFP- and the untransformed *C. trachomatis* SWFP-. These data are shown in Figure S5 and clearly demonstrate that acquisition of the transforming plasmid pGFP::SW2 for both *C. trachomatis* SWFP- and the plasmid-free LGV isolate *C. trachomatis* L2(25667R) increases the infectious yield.

### Deletion of CDS5 from the *C. trachomatis* Plasmid

**Figure 2:**
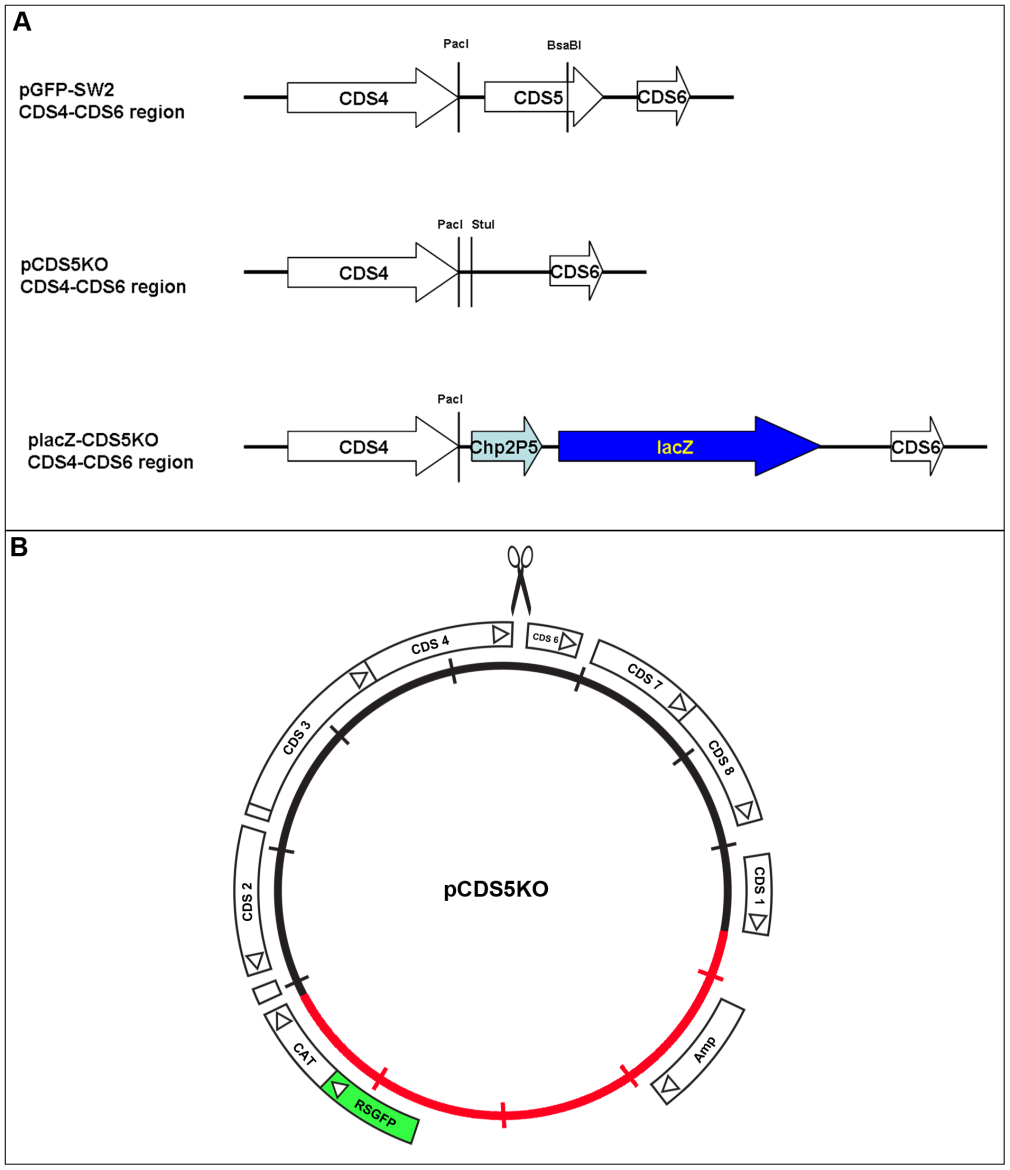
Construction of the CDS5 knock-out vector and insertion of the lacZ cassette. A. The regions between plasmid CDS4-CDS6 are shown, which highlight differences between the three plasmid constructs listed. In pCDS5KO, the ∼600 bp PacI-BsaBI fragment in pGFP::SW2 was replaced with a 35 bp oligo (including a unique StuI site for further cloning), resulting the deletion of CDS5. In placZ-CDS5KO, a *lacZ* cassette was inserted into the StuI site of pCDS5KO. This *lacZ* cassette contains a ∼200 bp promoter from chlamydiaphage Chp2 ORF5 (Chp2P5) placed in front of a 3.6 kb HindIII-PsiI *lacZ* fragment from pSV-B-Gal (Promega). The drawings are not to scale. B. Map showing the features of plasmid pCDS5KO. The inner circle represents the plasmid pSW2 from *C. trachomatis* SW2 (black) and the non-chlamydial sequences (red). The circle is graduated with 1 kb scale bars. The location of CDS 4 and CDS 6 are marked and the deletion of CDS5 is represented by the scissors symbol.

CDS5 is the most variable of the eight plasmid coding sequences and its gene product, pgp3, is secreted from the inclusion to the cell cytoplasm of *C. trachomatis* infected cells, thus this gene/gene product is unlikely to be involved in plasmid replication or maintenance. Hence, the open reading frame and promoter region for CDS5 were deleted from the shuttle vector pSW2::GFP by replacing the 655 bp fragment between *Pac*I and *Bsa*BI with a 35 bp oligonucleotide linker 5′-TAATAGCAAGCTTGAAACTAAAAACCAGGCCTGAT-3′ (containing a unique *Stu*I site for use in further cloning); this new plasmid is designated pCDS5KO (Figure 2, see Materials & Methods for the cloning details). Plasmid pCDS5KO was maintained in *E. coli* and then used to transform *C. trachomatis*.

Selection of penicillin resistant transformants of *C. trachomatis* SWFP- with pCDS5KO was straightforward (Figure S6A) and the transformed *C. trachomatis* inclusions fluoresced green (Figure S6B). In addition, inclusions stained positive for glycogen production (Figure S6C). These properties were maintained during passage of the transformants under penicillin selection indicating that the *C. trachomatis* SWFP- transformed by pCDS5KO was stable and thus CDS5 was not required for plasmid replication and maintenance.

### Construction of an Expression Cassette

We wanted to investigate whether it was possible to insert a large, functional gene into the chlamydial plasmid that itself conferred no selectable advantage on the host bacterium and could perhaps be useful in developing assays of promoter activity. Since lactose metabolism was the first system in which gene regulation was studied there are many genetic tools and assays that utilise the lactose operon, therefore we choose specifically to study the β-galactosidase gene/enzyme as proof of principle that such a construct would be stable and maintained. Furthermore, the *C. trachomatis* genome lacks the genes for the metabolism of lactose thus we could avoid any potential problems of dual functionality or recombination with the chlamydial chromosomal DNA. The β-galactosidase enzyme is also a useful test system for functionality as it is encoded by a large gene (∼3.5 kbp) made up of 4 homopolymeric subunits that have to assemble to form the active enzyme in the bacterial cytoplasm. β-galactosidase hydrolyzes lactose into glucose and galacatose, which is achieved by hydrolyzing the beta 1,4 glycosidic linkage between the two monosaccharides. We chose to use an expression cassette where a minimal chlamydiaphage promoter was fused in tandem to a β-galactosidase gene and this was cloned into the deleted CDS5 site of pCDS5KO to generate construct placZ-CDS5KO (Figure 2). Expression of β-galactosidase in *E.coli* was demonstrated by enzyme assay using X-gal in the culture medium as shown in Figure S7.

### Expression of β-galactosidase in *C. trachomatis*

**Figure 3:**
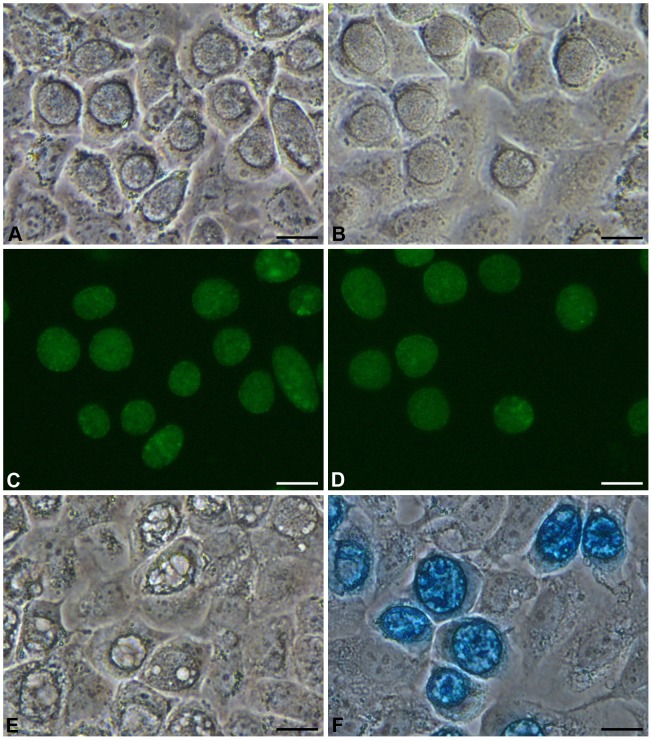
Properties of *C. trachomatis* SWFP^−^ transformed with plasmid pCDS5KO and placZ-CDS5KO. Microscopic images of live McCoy cells infected with *C. trachomatis* SWFP^−^ transformed by pCDS5KO (A) and placZ CDS5KO (B) under white light (phase contrast) and the same fields under blue light (C and D). Panels E and F show McCoy cells infected with *C. trachomatis* SWFP- transformed by pCDS5KO (E) and placZ-CDS5KO (F). The cells in these panels have been fixed with Formaldehyde and stained with X-gal. Active β-galactosidase cleaves X-gal to form an insoluble blue precipitate. Mature SWFP^−/^pCDS5KO and SWFP^−/^placZ CDS5KO inclusions are morphologically indistinguishable by phase contrast microscopy (A and B). The scale bar represents 20 µm.

McCoy cells infected with *C. trachomatis* SWFP−/pCDS5KO and *C. trachomatis* SWFP−/placZ-CDS5KO were grown under penicillin selection and then fixed and tested for β-galactosidase activity by X-gal staining. Inclusions formed by *C. trachomatis* SWFP−/placZ-CDS5KO stained intensely blue whereas inclusions formed by *C. trachomatis* SWFP−/pCDS5KO did not stain (Figure 3). These results demonstrate that the β-galactosidase is active in *C. trachomatis.*

Purified EBs and RBs from *C. trachomatis* transformed by pCDS5KO were assayed for plasmid copy number and they showed no significant differences to plasmid copy numbers compared with the wild type *C.trachomatis* from previous studies [27] (Figure S8).

## Discussion

A transformation protocol was recently developed for *C. trachomatis* L2/434/Bu which is a well-studied, laboratory-adapted isolate from the LGV biovar that grows rapidly and thus has been widely used around the world over the past 40 years for laboratory studies. However, LGV isolates are not typical of most *C. trachomatis* and whilst they are a useful model for studying many aspects of chlamydial cell and molecular microbiology we considered that it was important to apply the transformation technology to *C. trachomatis* belonging to the trachoma biovar. *C. trachomatis* isolates of the trachoma biovar (including genital tract strains) are generally slower growing than LGV isolates and require assistance to infect cells, this is usually achieved by centrifugation or the addition of cations such as DEAE cellulose. Preparations of the trachoma biovar isolates have much lower particle to infectivity ratios [38], perhaps an indication of their reduced virulence *in vivo*. For this project we have applied the plasmid transformation protocol to a Swedish genital tract isolate of *C. trachomatis* that has had little adaptation to cell culture. In our previous work selecting transformants with the LGV *C. trachomatis* isolate L2/434/Bu a key element of the experimental design was the need to perform multiple rounds of plaque purification to obtain clonal transformants. LGV isolates of *C. trachomatis* form large and easily reproducible plaques, by contrast plaquing of the less invasive, non-LGV *C. trachomatis* is a technically demanding and time consuming procedure. During the plaque purification process the endogenous chlamydial plasmid was eliminated from the *C. trachomatis* L2/434/Bu host, presumably by competition and incompatibility with the transforming vector under antibiotic selection. The Swedish *C. trachomatis* isolate (SWFP-) was selected as a potential recipient host as it is a naturally occurring plasmid-free strain and thus our experimental design, incorporating this isolate, meant plaque purification was not required. In this work we have demonstrated that the Calcium Chloride- based transformation protocol works with a genital tract isolate that has had very limited attenuation under laboratory conditions. Using the original plasmid vector pGFP::SW2 we have routinely and rapidly recovered transformants of *C. trachomatis* SWFP- with the modified protocol. These results showed that the use of a genital tract isolate as a recipient host, with the introduction of a centrifugation step into the standard transformation protocol, allowed the selection of transformants. Acquisition of the recombinant plasmid restores glycogen biosynthesis (a function associated with the plasmid in *C. trachomatis*) (Figure 1C’), and the transformants are penicillin resistant and express the green fluorescent protein (Figure 1B’). The additional advantage of using plasmid-free *C. trachomatis* as a recipient host is that it speeds up the process removing the need for plaque purification and eliminates the possibility of the transforming vector recombining with the endogenous plasmid.

We noticed that the pGFP::SW2 transformants grew faster than the untransformed recipient *C.trachomatis* (SWFP-), consistent with the evidence that the *C. trachomatis* plasmid is a virulence factor [39]. Previously we had also transformed a naturally occurring plasmid-free LGV isolate of *C. trachomatis* and this also appeared to grow more quickly once transformed by the plasmid pGFP::SW2. Thus plasmid-free *C. trachomatis* transformed with the recombinant plasmid, which brings a replicative burden, have an increased growth rate and infectious yield. It has been suggested that plasmid-free *C. trachomatis* might be useful as an attenuated vaccine because of its reduced virulence. Our *in vitro* data showing a more rapid growth characteristics provide supportive evidence that the plasmid has a role in *C. trachomatis* virulence. However, some caution may need to be exercised in such an approach to vaccination. The original clinical data from Sweden show the plasmid-free Swedish isolate retained its ability to infect *in vivo* as two plasmid-free ‘serovar F’ isolates were obtained, one from each adult in the couple, indicating that transmission of the plasmid-free *C. trachomatis* had occurred between partners. Also, as mixed infections occur, plasmids could be transferred back into plasmid deficient *C. trachomatis* isolates, restoring virulence.

To construct the original shuttle vector we had inserted *E. coli* components (origin of replication and selectable markers) within CDS1 as this region of the chlamydial plasmid is naturally mutable, hinting that the function of CDS1 was not essential. In the current study we wanted to investigate whether other regions of the chlamydial plasmid were dispensable and could be used for further insertion of ‘foreign’ DNA. For this purpose we chose CDS5 since the gene product, the protein pgp3, is secreted to the infected cell cytoplasm and forms a complex trimeric structure. Since this protein appears to have no role within EBs or RBs we considered that it was unlikely to be involved in plasmid maintenance or replication. We constructed a simple deletion mutant of CDS5 in the shuttle vector pSW2::GFP and transformation of the genital tract *C. trachomatis* SWFP- with this new construct pCDS5KO was possible proving that CDS5 is not an essential component of the plasmid, required for replication or maintenance. Plasmid free isolates of *Chlamydia* have two clear phenotypes, those with the inability to accumulate glycogen which demonstrate the production of ‘bull’s eye’ inclusions. The parental *C. trachomatis* SWFP- also exhibited both of these phenotypic traits. Recently we proved that acquisition of the chlamydial plasmid by an LGV, plasmid-free isolate restored both the ability to accumulate glycogen and to produce ‘normal’ inclusions and this was also the case for *C. trachomatis* SWFP- when transformed with the standard transformation vector pSW2::GFP. *C. trachomatis* SWFP- transformed with the pCDS5KO plasmid had restored both the ability to accumulate glycogen and normal inclusion morphology indicating that neither of these properties were related to the presence of CDS5. To verify that CDS5 has no role in plasmid maintenance and stability, the copy number of the CDS5 knockout and the starting vector were measured for both EBs and RBs, and found to be similar to that of wild-type genital tract isolates of *C. trachomatis* [27] indicating that CDS5 or its gene product pgp3 has no role in regulating plasmid copy number. To test whether the deleted region of the plasmid in CDS5 was a suitable cloning/insertion site for foreign DNA and to explore the possible size limit of a stable and functional insert we constructed a plasmid cassette based on pCDS5KO containing the *lac Z* gene. Transformation of *C. trachomatis* with this new plasmid, placZ-CDS5KO, showed that β-galactosidase was expressed as an active enzyme.

Thus, in conclusion, we have shown that it is possible to transform a genital tract isolate of *C. trachomatis* (that requires centrifugation to initiate cell infection). Acquisition of the plasmid increases the growth rate and thereby shortens the developmental cycle. However, in contrast to studies on *C. muridarum* where infectivity is significantly reduced in plasmid- cured isolates, plasmid bearing *C. trachomatis* still require centrifugation to assist infection. It is possible to engineer a knockout for the CDS5 and insert foreign DNA at this site in the plasmid. As proof of principle we have shown that it is possible to express functional β-galactosidase. Knock out of the CDS5 region has no effect on the plasmid-associated phenotypes of glycogen accumulation and the production of bull’s eye inclusions. The ability to express active β-galactosidase within chlamydial inclusions is an important advance as it opens the possibility to measure accurately promoter activity and to devise assays to assess effectors of promoter activity during the developmental cycle. This work has established that it is possible to selectively delete segments of the chlamydial plasmid; this is the first step towards a detailed molecular dissection of the role of the plasmid in regulating biochemical functions (e.g glycogen accumulation), biological functions (inclusion morphology) and virulence. The next steps in this work are to evaluate the effects of this gene knock out on infection *in vivo*.

## Supporting Information

Figure S1 **Plasmid pCDS5KO nucleotide sequence and features.** The pCDS5KO plasmid is a derivative of pGFP::SW2 and has the *C. trachomatis* plasmid CDS5 region deleted.
(DOC)

Figure S2 **Plasmid placZ-CDS5KO nucleotide sequence and features.** This plasmid has the *lacZ* gene under control of a tandem arrangement of the chlamydiaphage (Chp2) ORF5 promoter and a minimal *E. coli* gpt promoter.
(DOC)

Figure S3 **Microscopic images showing the early stages of recovery for transformants of** ***C. trachomatis*** **SWFP- with plasmid pGFP::SW2.** The transformation mix (*C. trachomatis* SWFP-, plasmid DNA and McCoy cells in CaCl_2_/Tris buffer) was seeded onto 6-well plate in penicillin-free medium for two days before harvest T_0_ (A). Recovery of transformants was performed by 2–3 rounds of passages under penicillin selection (10 units/ml) in McCoy cells in T_25_ flasks (B–F). After two days in Passage 1, all inclusions appeared to be ‘abnormal’ (B). Nevertheless, after two days in Passage 2, ‘normal’ inclusions begin to emerge (C&D, D is the same field as C under blue light). More transformants were grown in Passage 3 (E&F, F is the same filed as E under blue light). Scale bars: 40 µm in images A&B and 100 µm in C–F.
(TIF)

Figure S4 **Southern blot of** ***C. trachomatis*** **SWFP- (−p) and** ***C. trachomatis*** **SWFP- transformed by plasmid pGFP::SW2 (+p) using a DIG-labelled GFP probe.** Six chlamydial genomic DNA samples (∼0.5 µg DNA/lane) were loaded on 1% agarose gel in pairs together with HyperLadder I (5 µl) from BIOLINE (Cat No. BIO-33025). The agarose gel image was taken before DNA transfer (A). The DNA blot was hybridized with the GFP probe (B). The *Bam* HI digestion of pGFP::SW2 generated 3 fragments: 7169 bp, 2925 bp and 1445 bp (containing GFP probe sequence). The *Bgl* II digestion of pGFP::SW2 generated 4 fragments: 5555 bp, 3625 bp (containing the GFP probe sequence), 1693 bp and 666 bp. The Southern blot showed that the hybridization signals at expected positions in all SWFP−/pGFP::SW2 samples (uncut or digested); whilst no hybridization signal was detected in all SWFP- samples (uncut or digested) (B).
(TIF)

Figure S5 ***C. trachomatis SWFP-*** **and** ***C. trachomatis*** **L2 (25667R) grow faster and give a higher yield when transformed by pGFP::SW2.** (A) *C. trachomatis* SWFP- (black) and *C. trachomatis SWFP-* transformed with pGFP::SW2 (green*)* (B) *C. trachomatis* L2 (25667R) and C. *trachomatis* L2 (25667R) transformed with pGFP::SW2. McCoy cells in a 24 well tissue culture tray grown to confluence were infected with *C. trachomatis* at MOI = 1 and were cultured as described. The yield of *C. trachomatis* (IFU) per culture or single well is shown on the y – axis and time of sampling post infection is shown on the x –axis. The experiments were repeated in quadruplicate and standard error bars are shown for each sample point.
(TIF)

Figure S6 **Properties of** ***C. trachomatis*** **SWFP- transformed with plasmid pCDS5KO (SWFP−/pCDS5KO).** (A) Image of live McCoy cells infected with SWFP−/pCDS5KO under white light (phase contrast). (B) The same field as (A) under blue light. (C) Iodine stained McCoy cells (Methanol-fixed on coverslips) infected with SWFP−/pCDS5KO. The transformant SWFP−/pCDS5KO expressed the green fluorescent protein and was iodine-stain positive. Scale bar represents 20 µm.
(TIF)

Figure S7 **Phenotypic properties** ***E. coli*** **transformed with plasmids pCDS5KO and placZ-CDS5KO.** *E. coli* strain MC1061 transformed with pCDS5KO and placZ-CDS5KO was grown on (A) an LB-amp agar plate, and (B) LB-Xgal-amp agar plate. Expression of the green fluorescent protein was visualised under blue light.
(TIF)

Figure S8 **Plasmid copy number in** ***C. trachomatis*** **SWFP- transformed by pCDS5KO.** Genomic DNA was extracted from gradient-purified EBs and RBs and analysed by qPCR to determine the plasmid/genome ratios (P/G). Standard error bars are shown.
(TIF)
