# Supplementary material for: Genetic Transformation of a Clinical (Genital Tract), Plasmid-Free Isolate of Chlamydia trachomatis: Engineering the Plasmid as a Cloning Vector
Source: PLoS One. 2013 Mar 18;8(3):e59195. doi: 10.1371/journal.pone.0059195 (PMC3601068; doi:10.1371/journal.pone.0059195)
Supplement: Figure S1 — Plasmid pCDS5KO nucleotide sequence and features. The pCDS5KO plasmid is a derivative of pGFP::SW2 and has the C. trachomatis plasmid CDS5 region deleted. (DOC) [file pone.0059195.s001.doc]

**Figure S1 Plasmid pCDS5KO nucleotide sequence and features**

Plasmid pCDS5KO (10919 bp) was constructed from pGFP::SW2 (11539 bp) with the CDS5 promoter and ¾ of CDS5 coding region deleted. It was made by replacing the 1422bp EcoRI (in CDS4) and BsaBI (in CDS5) fragment in pGFP::SW2 with a 802bp EcoRI-EcoRV fragment from CDS4 PCR product, resulting in a 629 bp deletion from pGFP::SW2 (between the 24^th^ bp after CDS4 stop codon TAA and the BsaBI site in CDS5) and a 9 bp insertion (including a unique StuI site for further cloning). The clone was generated by ‘three-fragment ligation’: two restriction endonuclease cleavage fragments from pGFP::SW2 (the 8101 bp BsaBI fragment and the 2016 bp BsaBI-EcoRI fragment) and a 802bp EcoRI-EcoRV fragment from the CDS4 PCR product, using DNA template pGFP::SW2 and primers ORF4_F(RI) (5’-AAAACTGAATTCTTAGAGGCTTATGG-3’) and ORF4_R(RV) (5’-AAAAAAGATATCAGGCCTGGTTTTTAGTTTCAAGCTTGCTATTA-3’). The PCR region and the ligation sites of pCDS5KO were verified by sequence analysis.

| **Position on pCDS5KO** | **Feature** | **Length (bp)** | **Source** | **GenBank #**  **or Reference** |
| --- | --- | --- | --- | --- |
| 1-2900 | 1-2900 on pGFP::SW2 | 2900 | pGFP::SW2 | Wang et al, 2011 |
| 2901-3702 | 2901-3693 on pGFP::SW2 plus 9nt insertion AGGCCTGAT (with a unique StuI site) from primer ORF4_R(RV) | 802 | CDS4 PCR with primers ORF4_F(RI) & ORF4_R(RV) and template pGFP::SW2 | This paper |
| 3703-10919 | 4323-11539 on pGFP::SW2 | 7217 | pGFP::SW2 | Wang et al, 2011 |
|  |  |  |  |  |
| 1-12 | pSP73 SalI-BamHI fragment | 12 | pSP73 | X65333.2 |
| 13-3693 | pSW2 fragment from BamHI in CDS1 to the 23^rd^ nt after CDS4 stop codon TAA | 3681 | pSW2 | FM865439.1 |
| 122🡨1114 | CDS2 | 993 |  |  |
| 1257🡪2612 | CDS3 | 1356 |  |  |
| 2606🡪3670 | CDS4 | 1065 |  |  |
| 3694-3702 | 9nt insertion AGGCCTGAT | 9 | PCR primer ORF4_R(RV) | This paper |
| 3703-6561 | pSW2 fragment from BsaBI in CDS5 to BamHI in CDS1 | 2859 | pSW2 | FM865439.1 |
| 3976🡪4284 | CDS6 | 309 |  |  |
| 4375🡪5106 | CDS7 | 732 |  |  |
| 5103🡪5846 | CDS8 | 744 |  |  |
| 6080🡪6561 | CDS1 (part of) | 482 |  |  |
| 6562-9011 | pSP73 BamHI-PstI fragment | 2450 | pSP73 | X65333.2 |
| 7078🡪7938 | Amp | 861 |  |  |
| 8083-8756 | pUC ori | 674 |  |  |
| 9012-10919 | pRSGFPCAT PstI-SalI fragment | 1908 | pRSGFPCAT | Wang et al, 2011 |
| 9012🡪9486 | MCIP | 475 |  |  |
| 9502🡪10230 | RSGFP | 729 |  |  |
| 10231🡪10890 | CAT | 660 |  |  |

Sequence of pCDS5KO (sequences around cloning sites and PCR region were verified)

1 TCGACTCTAG AGGATCCGTT TGTTCTGGGG AAGAGGTAAT TCCTCTAGTA CAAACACCCA CAATATTGTG ATATAATTAA AATTATATTC ATATTCTGTT

101 GCCAGAAAAA ACACCTTTAG GCTATATTAG AGCCAGCTTC TTTGAAGCGT TGTCTTCTCG AGAAGATTTA TCGTACGCAA ATATCATCTT TGCGGTTGCG

201 TGTCCTGTGA CCTTCATTAT GTCGGAGTCT GAGCACCCTA GGCGTTTGTA CTCCGTCACA GCGGTTGCTC GAAGCACGTG CGGGGTTATT TTAAAAGGGA

301 TTGCAGCTTG TAGTCCTGCT TGAGAGAACG TGCGGGCGAT TTGCCTTAAC CCCACCATTT TTCCGGAGCG AGTTACGAAG ACAAAACCTC TTCGTTGACC

401 GATGTACTCT TGTAGAAAGT GCATAAACTT CTGAGGATAA GTTATAATAA TCCTCTTTTC TGTCTGACGG TTCTTAAGCT GGGAGAAAGA AATGGTAGCT

501 TGTTGGAAAC AAATCTGACT AATCTCCAAG CTTAAGACTT CAGAGGAGCG TTTACCTCCT TGGAGCATTG TCTGGGCGAT CAACCAATCC CGGGCATTGA

601 TTTTTTTTAG CTCTTTTAGG AAGGATGCTG TTTGCAAACT GTTCATCGCA TCCGTTTTTA CTATTTCCCT GGTTTTAAAA AATGTTCGAC TATTTTCTTG

701 TTTAGAAGGT TGCGCTATAG CGACTATTCC TTGAGTCATC CTGTTTAGGA ATCTTGTTAA GGAAATATAG CTTGCTGCTC GAACTTGTTT AGTACCTTCG

801 GTCCAAGAAG TCTTGGCAGA GGAAACTTTT TTAATCGCAT CTAGGATTAG ATTATGATTT AAAAGGGAAA ACTCTTGCAG ATTCATATCC AAGGACAATA

901 GACCAATCTT TTCTAAAGAC AAAAAAGATC CTCGATATGA TCTACAAGTA TGTTTGTTGA GTGATGCGGT CCAATGCATA ATAACTTCGA ATAAGGAGAA

1001 GCTTTTCATG CGTTTCCAAT AGGATTCTTG GCGAATTTTT AAAACTTCCT GATAAGACTT TTCACTATAT TCTAACGACA TTTCTTGCTG CAAAGATAAA

1101 ATCCCTTTAC CCATGAAATC CCTCGTGATA TAACCTATCC GTAAAATGTC CTGATTAGTG AAATAATCAG GTTGTTAACA GGATAGCACG CTCGGTATTT

1201 TTTTATATAA ACAGGTTGTT AACAGGATAG CACGCTCGGT ATTTTTTTAT ATAAACATGA AAACTCGTTC CGAAATAGAA AATCGCATGC AAGATATCGA

1301 GTATGCGTTG TTAGGTAAAG CTCTGATATT TGAAGACTCT ACTGAGTATA TTCTGAGGCA GCTTGCTAAT TATGAGTTTA AGTGTTCTCA TCATAAAAAC

1401 ATATTCATAG TATTTAAATA CTTAAAAGAC AATGGATTAC CTATAACTGT AGACTCGGCT TGGGAAGAGC TTTTGCGGCG TCGTATCAAA GATATGGACA

1501 AATCGTATCT CGGGTTAATG TTGCATGATG CTTTATCAAA TGACAAGCTT AGATCCGTTT CTCATACGGT TTTCCTCGAT GATTTGAGCG TGTGTAGCGC

1601 TGAAGAAAAT TTGAGTAATT TCATTTTCCG CTCGTTTAAT GAGTACAATG AAAATCCATT GCGTAGATCT CCGTTTCTAT TGCTTGAGCG TATAAAGGGA

1701 AGGCTTGATA GTGCTATAGC AAAGACTTTT TCTATTCGCA GCGCTAGAGG CCGGTCTATT TATGATATAT TCTCACAGTC AGAAATTGGA GTGCTGGCTC

1801 GTATAAAAAA AAGACGAGTA GCGTTCTCTG AGAATCAAAA TTCTTTCTTT GATGGCTTCC CAACAGGATA CAAGGATATT GATGATAAAG GAGTTATCTT

1901 AGCTAAAGGT AATTTCGTGA TTATAGCAGC TAGACCATCT ATAGGGAAAA CAGCTTTAGC TATAGACATG GCGATAAATC TTGCGGTTAC TCAACAGCGT

2001 AGAGTTGGTT TCCTATCTCT AGAAATGAGC GCAGGTCAAA TTGTTGAGCG GATTATTGCT AATTTAACAG GAATATCTGG TGAAAAATTA CAAAGAGGGG

2101 ATCTCTCTAA AGAAGAATTA TTCCGAGTAG AAGAAGCTGG AGAAACGGTT AGAGAATCAC ATTTTTATAT CTGCAGTGAT AGTCAGTATA AGCTTAACTT

2201 AATCGCGAAT CAGATCCGGT TGCTGAGAAA AGAAGATCGA GTAGACGTAA TATTTATCGA TTACTTGCAG TTGATCAACT CATCGGTTGG AGAAAATCGT

2301 CAAAATGAAA TAGCAGATAT ATCTAGAACC TTAAGAGGTT TAGCCTCAGA GCTAAACATT CCTATAGTTT GTTTATCCCA ACTATCTAGA AAAGTTGAGG

2401 ATAGAGCAAA TAAAGTTCCC ATGCTTTCAG ATTTGCGAGA CAGCGGTCAA ATAGAGCAAG ACGCAGATGT GATTTTGTTT ATCAATAGGA AGGAATCGTC

2501 TTCTAATTGT GAGATAACTG TTGGGAAAAA TAGACATGGA TCGGTTTTCT CTTCGGTATT ACATTTCGAT CCAAAAATTA GTAAATTCTC CGCTATTAAA

2601 AAAGTATGGT AAATTATAGT AACTGCCACT TCATCAAAAG TCCTATCCAC CTTGAAAATC AGAAGTTTGG AAGAAGACCT GGTCAATCTA TTAAGATATC

2701 TCCCAAATTG GCTCAAAATG GGATGGTAGA AGTTATAGGT CTTGATTTTC TTTCATCTCA TTACCATGCA TTAGCAGCTA TCCAAAGATT ACTGACCGCA

2801 ACGAATTACA AGGGGAACAC AAAAGGGGTT GTTTTATCCA GAGAATCAAA TAGTTTTCAA TTTGAAGGAT GGATACCAAG AATCCGTTTT ACAAAAACTG

2901 AATTCTTAGA GGCTTATGGA GTTAAGCGGT ATAAAACATC CAGAAATAAG TATGAGTTTA GTGGAAAAGA AGCTGAAACT GCTTTAGAAG CCTTATACCA

3001 TTTAGGACAT CAACCGTTTT TAATAGTGGC AACTAGAACT CGATGGACTA ATGGAACACA AATAGTAGAC CGTTACCAAA CTCTTTCTCC GATCATTAGG

3101 ATTTACGAAG GATGGGAAGG TTTAACTGAC GAAGAAAATA TAGATATAGA CTTAACACCT TTTAATTCAC CACCTACACG GAAACATAAA GGGTTCGTTG

3201 TAGAGCCATG TCCTATCTTG GTAGATCAAA TAGAATCCTA CTTTGTAATC AAGCCTGCAA ATGTATACCA AGAAATAAAA ATGCGTTTCC CAAATGCATC

3301 AAAGTATGCT TACACATTTA TCGACTGGGT GATTACAGCA GCTGCGAAAA AGAGACGAAA ATTAACTAAG GATAATTCTT GGCCAGAAAA CTTGTTATTA

3401 AACGTTAACG TTAAAAGTCT TGCATATATT TTAAGGATGA ATCGGTACAT CTGTACAAGG AACTGGAAAA AAATCGAGTT AGCTATCGAT AAATGTATAG

3501 AAATCGCCAT TAAGCTTGGC TGGTTATCTA GAAGAAAACG CATTGAATTT CTGGATTCTT CTAAACTCTC TAAAAAAGAA ATTCTATATC TAAATAAAGA

3601 GCGCTTTGAA GAAATAACTA AGAAATCTAA AGAACAAATG GAACAATTAG AACAAGAATC TATTAATTAA TAGCAAGCTT GAAACTAAAA ACCAGGCCTG

3701 ATTCATCAGG CATTCCTAAT TTATGTAGTC TAAGAACCAG TATTACTAAT ACAGGATTGA CTCCGACAAC GTATTCATTA CGTGTAGGCG GTTTAGAAAG

3801 CGGTGTGGTA TGGGTTAATG CCCTTTCTAA TGGCAATGAT ATTTTAGGAA TAACAAATAC TTCTAATGTA TCTTTTTTAG AGGTAATACC TCAAACAAAC

3901 GCTTAAACAA TTTTTATTGG ATTTTTCTTA TAGGTTTTAT ATTTAGAGAA AACAGTTCGA ATTACGGGGT TTGTTATGCA AAATAAAAGA AAAGTGAGGG

4001 ACGATTTTAT TAAAATTGTT AAAGATGTGA AAAAAGATTT CCCCGAATTA GACCTAAAAA TACGAGTAAA CAAGGAAAAA GTAACTTTCT TAAATTCTCC

4101 CTTAGAACTC TACCATAAAA GTGTCTCACT AATTCTAGGA CTGCTTCAAC AAATAGAAAA CTCTTTAGGA TTATTCCCAG ACTCTCCTGT TCTTGAAAAA

4201 TTAGAGGATA ACAGTTTAAA GCTAAAAAAG GCTTTGATTA TGCTTATCTT GTCTAGAAAA GACATGTTTT CCAAGGCTGA ATAGACAACT TACTCTAACG

4301 TTGGAGTTGA TTTGCACACC TTAGTTTTTT GCTCTTTTAA GGGAGGAACT GGAAAAACAA CACTTTCTCT AAACGTGGGA TGCAACTTGG CCCAATTTTT

4401 AGGGAAAAAA GTGTTACTTG CTGACCTAGA CCCGCAATCC AATTTATCTT CTGGATTGGG GGCTAGTGTC AGAAGTGACC AAAAAGGCTT GCACGACATA

4501 GTATACACAT CAAACGATTT AAAATCAATC ATTTGCGAAA CAAAAAAAGA TAGTGTGGAC CTAATTCCTG CATCATTTTC ATCCGAACAG TTTAGAGAAT

4601 TGGATATTCA TAGAGGACCT AGTAACAACT TAAAGTTATT TCTGAATGAG TACTGCGCTC CTTTTTATGA CATCTGCATA ATAGACACTC CACCTAGCCT

4701 AGGAGGGTTA ACGAAAGAAG CTTTTGTTGC AGGAGACAAA TTAATTGCTT GTTTAACTCC AGAACCTTTT TCTATTCTAG GGTTACAAAA GATACGTGAA

4801 TTCTTAAGTT CGGTCGGAAA ACCTGAAGAA GAACACATTC TTGGAATAGC TTTGTCTTTT TGGGATGATC GTAACTCGAC TAACCAAATG TATATAGACA

4901 TTATCGAGTC TATTTACAAA AACAAGCTTT TTTCAACAAA AATTCGTCGA GATATTTCTC TCAGCCGTTC TCTTCTTAAA GAAGATTCTG TAGCTAATGT

5001 CTATCCAAAT TCTAGGGCCG CAGAAGATAT TCTGAAGTTA ACGCATGAAA TAGCAAATAT TTTGCATATC GAATATGAAC GAGATTACTC TCAGAGGACA

5101 ACGTGAACAA ACTAAAAAAA GAAGCGGATG TCTTTTTTAA AAAAAATCAA ACTGCCGCTT CTCTAGATTT TAAGAAGACG CTTCCCTCCA TTGAACTATT

5201 CTCAGCAACT TTGAATTCTG AGGAAAGTCA GAGTTTGGAT CGATTATTTT TATCAGAGTC CCAAAACTAT TCGGATGAAG AATTTTATCA AGAAGACATC

5301 CTAGCGGTAA AACTGCTTAC TGGTCAGATA AAATCCATAC AGAAGCAACA CGTACTTCTT TTAGGAGAAA AAATCTATAA TGCTAGAAAA ATCCTGAGTA

5401 AGGATCACTT CTCCTCAACA ACTTTTTCAT CTTGGATAGA GTTAGTTTTT AGAACTAAGT CTTCTGCTTA CAATGCTCTT GCATATTACG AGCTTTTTAT

5501 AAACCTCCCC AACCAAACTC TACAAAAAGA GTTTCAATCG ATCCCCTATA AATCCGCATA TATTTTGGCC GCTAGAAAAG GCGATTTAAA AACCAAGGTC

5601 GATGTGATAG GGAAAGTATG TGGAATGTCG AACTCATCGG CGATAAGGGT GTTGGATCAA TTTCTTCCTT CATCTAGAAA CAAAGACGTT AGAGAAACGA

5701 TAGATAAGTC TGATTCAGAG AAGAATCGCC AATTATCTGA TTTCTTAATA GAGATACTTC GCATCATGTG TTCCGGAGTT TCTTTGTCCT CCTATAACGA

5801 AAATCTTCTA CAACAGCTTT TTGAACTTTT TAAGCAAAAG AGCTGATCCT CCGTCAGCTC ATATATATAT ATCTATTATA TATATATATT TAGGGATTTG

5901 ATTTCACGAG AGAGATTTGC AACTCTTGGT GGTAGACTTT GCAACTCTTG GTGGTAGACT TTGCAACTCT TGGTGGTAGA CTTTGCAACT CTTGGTGGTA

6001 GACTTGGTCA TAATGGACTT TTGTTAAAAA ATTTCTTAAA ATCTTAGAGC TCCGATTTTG AATAGCTTTG GTTAAGAAAA TGGGCTCGAT GGCTTTCCAT

6101 AAAAGTAGAT TGTTTTTAAC TTTTGGGGAC GCGTCGGAAA TTTGGTTATC TACTTTATCT TATCTAACTA GAAAAAATTA TGCGTCTGGG ATTAACTTTC

6201 TTGTTTCTTT AGAGATTCTG GATTTATCGG AAACCTTGAT AAAGGCTATT TCTCTTGACC ACAGCGAATC TTTGTTTAAA ATCAAGTCTC TAGATGTTTT

6301 TAATGGAAAA GTTGTTTCAG AGGCATCTAA ACAGGCTAGA GCGGCATGCT ACATATCTTT CACAAAGTTT TTGTATAGAT TGACCAAGGG ATATATTAAA

6401 CCCGCTATTC CATTGAAAGA TTTTGGAAAC ACTACATTTT TTAAAATCCG AGACAAAATC AAAACAGAAT CGATTTCTAA GCAGGAATGG ACAGTTTTTT

6501 TTGAAGCGCT CCGGATAGTG AATTATAGAG ACTATTTAAT CGGTAAATTG ATTGTACAAG GGATCCCCGG GTACCGAGCT CGAATTCATC GATGATATCA

6601 GATCTGGTTC TATAGTGTCA CCTAAATCGT ATGTGTATGA TACATAAGGT TATGTATTAA TTGTAGCCGC GTTCTAACGA CAATATGTCC ATATGGTGCA

6701 CTCTCAGTAC AATCTGCTCT GATGCCGCAT AGTTAAGCCA GCCCCGACAC CCGCCAACAC CCGCTGACGC GCCCTGACGG GCTTGTCTGC TCCCGGCATC

6801 CGCTTACAGA CAAGCTGTGA CCGTCTCCGG GAGCTGCATG TGTCAGAGGT TTTCACCGTC ATCACCGAAA CGCGCGAGAC GAAAGGGCCT CGTGATACGC

6901 CTATTTTTAT AGGTTAATGT CATGATAATA ATGGTTTCTT AGACGTCAGG TGGCACTTTT CGGGGAAATG TGCGCGGAAC CCCTATTTGT TTATTTTTCT

7001 AAATACATTC AAATATGTAT CCGCTCATGA GACAATAACC CTGATAAATG CTTCAATAAT ATTGAAAAAG GAAGAGTATG AGTATTCAAC ATTTCCGTGT

7101 CGCCCTTATT CCCTTTTTTG CGGCATTTTG CCTTCCTGTT TTTGCTCACC CAGAAACGCT GGTGAAAGTA AAAGATGCTG AAGATCAGTT GGGTGCACGA

7201 GTGGGTTACA TCGAACTGGA TCTCAACAGC GGTAAGATCC TTGAGAGTTT TCGCCCCGAA GAACGTTTTC CAATGATGAG CACTTTTAAA GTTCTGCTAT

7301 GTGGCGCGGT ATTATCCCGT ATTGACGCCG GGCAAGAGCA ACTCGGTCGC CGCATACACT ATTCTCAGAA TGACTTGGTT GAGTACTCAC CAGTCACAGA

7401 AAAGCATCTT ACGGATGGCA TGACAGTAAG AGAATTATGC AGTGCTGCCA TAACCATGAG TGATAACACT GCGGCCAACT TACTTCTGAC AACGATCGGA

7501 GGACCGAAGG AGCTAACCGC TTTTTTGCAC AACATGGGGG ATCATGTAAC TCGCCTTGAT CGTTGGGAAC CGGAGCTGAA TGAAGCCATA CCAAACGACG

7601 AGCGTGACAC CACGATGCCT GTAGCAATGG CAACAACGTT GCGCAAACTA TTAACTGGCG AACTACTTAC TCTAGCTTCC CGGCAACAAT TAATAGACTG

7701 GATGGAGGCG GATAAAGTTG CAGGACCACT TCTGCGCTCG GCCCTTCCGG CTGGCTGGTT TATTGCTGAT AAATCTGGAG CCGGTGAGCG TGGGTCTCGC

7801 GGTATCATTG CAGCACTGGG GCCAGATGGT AAGCCCTCCC GTATCGTAGT TATCTACACG ACGGGGAGTC AGGCAACTAT GGATGAACGA AATAGACAGA

7901 TCGCTGAGAT AGGTGCCTCA CTGATTAAGC ATTGGTAACT GTCAGACCAA GTTTACTCAT ATATACTTTA GATTGATTTA AAACTTCATT TTTAATTTAA

8001 AAGGATCTAG GTGAAGATCC TTTTTGATAA TCTCATGACC AAAATCCCTT AACGTGAGTT TTCGTTCCAC TGAGCGTCAG ACCCCGTAGA AAAGATCAAA

8101 GGATCTTCTT GAGATCCTTT TTTTCTGCGC GTAATCTGCT GCTTGCAAAC AAAAAAACCA CCGCTACCAG CGGTGGTTTG TTTGCCGGAT CAAGAGCTAC

8201 CAACTCTTTT TCCGAAGGTA ACTGGCTTCA GCAGAGCGCA GATACCAAAT ACTGTCCTTC TAGTGTAGCC GTAGTTAGGC CACCACTTCA AGAACTCTGT

8301 AGCACCGCCT ACATACCTCG CTCTGCTAAT CCTGTTACCA GTGGCTGCTG CCAGTGGCGA TAAGTCGTGT CTTACCGGGT TGGACTCAAG ACGATAGTTA

8401 CCGGATAAGG CGCAGCGGTC GGGCTGAACG GGGGGTTCGT GCACACAGCC CAGCTTGGAG CGAACGACCT ACACCGAACT GAGATACCTA CAGCGTGAGC

8501 ATTGAGAAAG CGCCACGCTT CCCGAAGGGA GAAAGGCGGA CAGGTATCCG GTAAGCGGCA GGGTCGGAAC AGGAGAGCGC ACGAGGGAGC TTCCAGGGGG

8601 AAACGCCTGG TATCTTTATA GTCCTGTCGG GTTTCGCCAC CTCTGACTTG AGCGTCGATT TTTGTGATGC TCGTCAGGGG GGCGGAGCCT ATGGAAAAAC

8701 GCCAGCAACG CGGCCTTTTT ACGGTTCCTG GCCTTTTGCT GGCCTTTTGC TCACATGTTC TTTCCTGCGT TATCCCCTGA TTCTGTGGAT AACCGTATTA

8801 CCGCCTTTGA GTGAGCTGAT ACCGCTCGCC GCAGCCGAAC GACCGAGCGC AGCGAGTCAG TGAGCGAGGA AGCGGAAGAG CGCCCAATAC GCAAACCGCC

8901 TCTCCCCGCG CGTTGGCCGA TTCATTAATG CAGGTTAACC TGGCTTATCG AAATTAATAC GACTCACTAT AGGGAGACCG GCCTCGAGCA GCTGAAGCTT

9001 GCATGCCTGC AGATGCCCGA CGGTCTTTAT AGCGGATTAA CAAAAATCAG GACAAGGCGG CGAAGCCGAA GACAGTACAA ATAGCACGGA ACCGATTCAC

9101 TTGGTGCTTC AGCACCTTAG AGAATCGTTC TCTTTGAGCT AAGGCGAGGC AACGCCGTAC TTGTTTTTGT TAATCCACTA TAAAGTGCCG CGTGTGTTTT

9201 TTTATGGCGT TTTAAAAAGC CGAGACTGCA TCCGGGCAGC AGCGCATCGG CCCGCACGAG GTCTGCGCTT GAATTGTGTT GTAGAAACAC AACGTTTTTT

9301 GAAAAAATAA GCTATTGTTT TATATCAAAA TATAATCATT TTTAAAATAA AGGTTGCGGC ATTTATCAGA TATTTGTTCT GAAAAATGGT TTTTTGCGGG

9401 GGGGGGGGTA TAATTGAAGA CGTATCGGGT GTTTGCCCGA TGTTTTTAGG TTTTTATCAA ATTTACAAAA GGAAGCCGAT ATGGTGGATC CCCGGGTACC

9501 AATGAGTAAA GGAGAAGCAC TTTTCACTGG AGTTGTCCCA ATTCTTGTTG AATTAGATGG TGATGTTAAT GGGCACAAAT TTTCTGTCAG TGGAGAGGGT

9601 GAAGGTGATG CAACATACGG AAAACTTACC CTTAAATTTA TTTGCACTAC TGGAAAACTA CCTGTTCCAT GGCCAACACT TGTCACTACT CTTACGTATG

9701 GTGTTCAATG CTTTTCAAGA TACCCAGATC ATATGAAACG GCATGACTTT TTCAAGAGTG CCATGCCCGA AGGTTATGTA CAGGAAAGAA CTATATTTTT

9801 CAAAGATGAC GGGAACTACA AGACACGTGC TGAAGTCAAG TTTGAAGGTG ATACCCTTGT TAATAGAATC GAGTTAAAAG GTATTGATTT TAAAGAAGAT

9901 GGAAACATTC TTGGACACAA ATTGGAATAC AACTATAACT CACACAATGT ATACATCATG GCAGACAAAC AAAAGAATGG AATCAAAGTT AACTTCAAAA

10001 TTAGACACAA CATTGAAGAT GGAAGCGTTC AACTAGCAGA CCATTATCAA CAAAATACTC CAATTGGCGA TGGCCCTGTC CTTTTACCAG ACAACCATTA

10101 CCTGTCCACA CAATCTGCCC TTTCGAAAGA TCCCAACGAA AAGAGAGACC ACATGGTCCT TCTTGAGTTT GTAACAGCTG CTGGGATTAC ACATGGCATG

10201 GATGAACTAT ACAAGTCCGG ACTCAGATCT ATGGAGAAAA AAATCACTGG ATATACCACC GTTGATATAT CCCAATGGCA TCGCAAAGAA CATTTTGAGG

10301 CATTTCAGTC AGTTGCTCAA TGTACCTATA ACCAGACCGT TCAGCTGGAT ATTACGGCCT TTTTAAAGAC CGTAAAGAAA AATAAGCACA AGTTTTATCC

10401 GGCCTTTATT CACATTCTTG CCCGCCTGAT GAATGCTCAT CCGGAATTCC GTATGGCAAT GAAAGACGGT GAGCTGGTGA TATGGGATAG TGTTCACCCT

10501 TGTTACACCG TTTTCCATGA GCAAACTGAA ACGTTTTCAT CGCTCTGGAG TGAATACCAC GACGATTTCC GGCAGTTTCT ACACATATAT TCGCAAGATG

10601 TGGCGTGTTA CGGTGAAAAC CTGGCCTATT TCCCTAAAGG GTTTATTGAG AATATGTTTT TCGTCTCAGC CAATCCCTGG GTGAGTTTCA CCAGTTTTGA

10701 TTTAAACGTG GCCAATATGG ACAACTTCTT CGCCCCCGTT TTCACCATGG GCAAATATTA TACGCAAGGC GACAAGGTGC TGATGCCGCT GGCGATTCAG

10801 GTTCATCATG CCGTTTGTGA TGGCTTCCAT GTCGGCAGAA TGCTTAATGA ATTACAACAG TACTGCGATG AGTGGCAGGG CGGGGCGTAA AGATCTCGAG

10901 CTCGATATCT AGATTAATG
