# Supplementary material for: Genetic Transformation of a Clinical (Genital Tract), Plasmid-Free Isolate of Chlamydia trachomatis: Engineering the Plasmid as a Cloning Vector
Source: PLoS One. 2013 Mar 18;8(3):e59195. doi: 10.1371/journal.pone.0059195 (PMC3601068; doi:10.1371/journal.pone.0059195)
Supplement: Figure S2 — Plasmid placZ-CDS5KO nucleotide sequence and features. This plasmid has the lacZ gene under control of a tandem arrangement of the chlamydiaphage (Chp2) ORF5 promoter and a minimal E. coli gpt promoter. (DOC) [file pone.0059195.s002.doc]

**Figure S2 Plasmid placZ-CDS5KO nucleotide sequence and features**

Plasmid placZ-CDS5KO (14774 bp) was constructed by cloning a modified *lacZ* cassette into the unique StuI site in pCDS5KO. The *lacZ* cassette contains a ~200bp promoter from chlamydiaphage Chp2 (NC_002194) ORF5 (Chp2P5) in tandem with the minimal promoter for *lacZ* from the vector pSV-B-Gal (Promega). placZ-CDS5KO was constructed in two steps: firstly, the 226bp BamHI-HindIII fragment of Chp2P5 and the 3749bp HindIII-SalI fragment of *lacZ* from pSV-B-Gal (Promega) were cloned into the BamHI and XhoI sites of pSP73 to get an intermediate plasmid pSP73-Chp2P5-lacZ; secondly, the 3855bp Smal-PsiI fragment from pSP73-Chp2P5-lacZ (the *lacZ* cassette) was inserted into the unique StuI site of pCDS5KO to generate the final plasmid placZ-CDS5KO. The PCR region and the ligation sites of placZ-CDS5KO were verified by sequence analysis.

| **Position on**  **placZ-CDS5KO** | **Feature** | **Length (bp)** | **Source** | **GenBank #**  **or Reference** |
| --- | --- | --- | --- | --- |
| 1-3696 | 1-3696 on pCDS5KO | 3696 | pCDS5KO | This paper  (see Figure S1) |
| 3697-7551 | Insertion of the Chp2P5-lacZ cassette  (SmaI-PsiI from pSP73-Chp2P5-lacZ) | 3855 | pSP73-Chp2P5-lacZ | This paper  (see below) |
| 3697-3699 | SmaI-BamHI from pSP73 | 3 | pSP73 | X65333.2 |
| 3700-3925 | BamHI-HindIII from Chp2P5 PCR | 226 | Chp2P5 PCR |  |
| 3702🡪3925 | Chp2P5 (promoter of Chp2 ORF5 (CDS4) ) | 224 | Chp2 (4083-4306) | NC_002194 |
| 3926-7551 | HindIII-PsiI from pSV-B-Gal (Promega) | 3626 | pSV-B-Gal (227-3852) | pSV-B-Gal |
| 4221🡪7268 | lacZ | 3048 | pSV-B-Gal (522-3569) | pSV-B-Gal |
| 7552-14774 | 3697-10919 on pCDS5KO | 7223 | pCDS5KO | This paper  (see Figure S1) |

Sequence of placZ-CDS5KO (sequences around cloning sites and PCR region were verified)

1 TCGACTCTAG AGGATCCGTT TGTTCTGGGG AAGAGGTAAT TCCTCTAGTA CAAACACCCA CAATATTGTG ATATAATTAA AATTATATTC ATATTCTGTT

101 GCCAGAAAAA ACACCTTTAG GCTATATTAG AGCCAGCTTC TTTGAAGCGT TGTCTTCTCG AGAAGATTTA TCGTACGCAA ATATCATCTT TGCGGTTGCG

201 TGTCCTGTGA CCTTCATTAT GTCGGAGTCT GAGCACCCTA GGCGTTTGTA CTCCGTCACA GCGGTTGCTC GAAGCACGTG CGGGGTTATT TTAAAAGGGA

301 TTGCAGCTTG TAGTCCTGCT TGAGAGAACG TGCGGGCGAT TTGCCTTAAC CCCACCATTT TTCCGGAGCG AGTTACGAAG ACAAAACCTC TTCGTTGACC

401 GATGTACTCT TGTAGAAAGT GCATAAACTT CTGAGGATAA GTTATAATAA TCCTCTTTTC TGTCTGACGG TTCTTAAGCT GGGAGAAAGA AATGGTAGCT

501 TGTTGGAAAC AAATCTGACT AATCTCCAAG CTTAAGACTT CAGAGGAGCG TTTACCTCCT TGGAGCATTG TCTGGGCGAT CAACCAATCC CGGGCATTGA

601 TTTTTTTTAG CTCTTTTAGG AAGGATGCTG TTTGCAAACT GTTCATCGCA TCCGTTTTTA CTATTTCCCT GGTTTTAAAA AATGTTCGAC TATTTTCTTG

701 TTTAGAAGGT TGCGCTATAG CGACTATTCC TTGAGTCATC CTGTTTAGGA ATCTTGTTAA GGAAATATAG CTTGCTGCTC GAACTTGTTT AGTACCTTCG

801 GTCCAAGAAG TCTTGGCAGA GGAAACTTTT TTAATCGCAT CTAGGATTAG ATTATGATTT AAAAGGGAAA ACTCTTGCAG ATTCATATCC AAGGACAATA

901 GACCAATCTT TTCTAAAGAC AAAAAAGATC CTCGATATGA TCTACAAGTA TGTTTGTTGA GTGATGCGGT CCAATGCATA ATAACTTCGA ATAAGGAGAA

1001 GCTTTTCATG CGTTTCCAAT AGGATTCTTG GCGAATTTTT AAAACTTCCT GATAAGACTT TTCACTATAT TCTAACGACA TTTCTTGCTG CAAAGATAAA

1101 ATCCCTTTAC CCATGAAATC CCTCGTGATA TAACCTATCC GTAAAATGTC CTGATTAGTG AAATAATCAG GTTGTTAACA GGATAGCACG CTCGGTATTT

1201 TTTTATATAA ACAGGTTGTT AACAGGATAG CACGCTCGGT ATTTTTTTAT ATAAACATGA AAACTCGTTC CGAAATAGAA AATCGCATGC AAGATATCGA

1301 GTATGCGTTG TTAGGTAAAG CTCTGATATT TGAAGACTCT ACTGAGTATA TTCTGAGGCA GCTTGCTAAT TATGAGTTTA AGTGTTCTCA TCATAAAAAC

1401 ATATTCATAG TATTTAAATA CTTAAAAGAC AATGGATTAC CTATAACTGT AGACTCGGCT TGGGAAGAGC TTTTGCGGCG TCGTATCAAA GATATGGACA

1501 AATCGTATCT CGGGTTAATG TTGCATGATG CTTTATCAAA TGACAAGCTT AGATCCGTTT CTCATACGGT TTTCCTCGAT GATTTGAGCG TGTGTAGCGC

1601 TGAAGAAAAT TTGAGTAATT TCATTTTCCG CTCGTTTAAT GAGTACAATG AAAATCCATT GCGTAGATCT CCGTTTCTAT TGCTTGAGCG TATAAAGGGA

1701 AGGCTTGATA GTGCTATAGC AAAGACTTTT TCTATTCGCA GCGCTAGAGG CCGGTCTATT TATGATATAT TCTCACAGTC AGAAATTGGA GTGCTGGCTC

1801 GTATAAAAAA AAGACGAGTA GCGTTCTCTG AGAATCAAAA TTCTTTCTTT GATGGCTTCC CAACAGGATA CAAGGATATT GATGATAAAG GAGTTATCTT

1901 AGCTAAAGGT AATTTCGTGA TTATAGCAGC TAGACCATCT ATAGGGAAAA CAGCTTTAGC TATAGACATG GCGATAAATC TTGCGGTTAC TCAACAGCGT

2001 AGAGTTGGTT TCCTATCTCT AGAAATGAGC GCAGGTCAAA TTGTTGAGCG GATTATTGCT AATTTAACAG GAATATCTGG TGAAAAATTA CAAAGAGGGG

2101 ATCTCTCTAA AGAAGAATTA TTCCGAGTAG AAGAAGCTGG AGAAACGGTT AGAGAATCAC ATTTTTATAT CTGCAGTGAT AGTCAGTATA AGCTTAACTT

2201 AATCGCGAAT CAGATCCGGT TGCTGAGAAA AGAAGATCGA GTAGACGTAA TATTTATCGA TTACTTGCAG TTGATCAACT CATCGGTTGG AGAAAATCGT

2301 CAAAATGAAA TAGCAGATAT ATCTAGAACC TTAAGAGGTT TAGCCTCAGA GCTAAACATT CCTATAGTTT GTTTATCCCA ACTATCTAGA AAAGTTGAGG

2401 ATAGAGCAAA TAAAGTTCCC ATGCTTTCAG ATTTGCGAGA CAGCGGTCAA ATAGAGCAAG ACGCAGATGT GATTTTGTTT ATCAATAGGA AGGAATCGTC

2501 TTCTAATTGT GAGATAACTG TTGGGAAAAA TAGACATGGA TCGGTTTTCT CTTCGGTATT ACATTTCGAT CCAAAAATTA GTAAATTCTC CGCTATTAAA

2601 AAAGTATGGT AAATTATAGT AACTGCCACT TCATCAAAAG TCCTATCCAC CTTGAAAATC AGAAGTTTGG AAGAAGACCT GGTCAATCTA TTAAGATATC

2701 TCCCAAATTG GCTCAAAATG GGATGGTAGA AGTTATAGGT CTTGATTTTC TTTCATCTCA TTACCATGCA TTAGCAGCTA TCCAAAGATT ACTGACCGCA

2801 ACGAATTACA AGGGGAACAC AAAAGGGGTT GTTTTATCCA GAGAATCAAA TAGTTTTCAA TTTGAAGGAT GGATACCAAG AATCCGTTTT ACAAAAACTG

2901 AATTCTTAGA GGCTTATGGA GTTAAGCGGT ATAAAACATC CAGAAATAAG TATGAGTTTA GTGGAAAAGA AGCTGAAACT GCTTTAGAAG CCTTATACCA

3001 TTTAGGACAT CAACCGTTTT TAATAGTGGC AACTAGAACT CGATGGACTA ATGGAACACA AATAGTAGAC CGTTACCAAA CTCTTTCTCC GATCATTAGG

3101 ATTTACGAAG GATGGGAAGG TTTAACTGAC GAAGAAAATA TAGATATAGA CTTAACACCT TTTAATTCAC CACCTACACG GAAACATAAA GGGTTCGTTG

3201 TAGAGCCATG TCCTATCTTG GTAGATCAAA TAGAATCCTA CTTTGTAATC AAGCCTGCAA ATGTATACCA AGAAATAAAA ATGCGTTTCC CAAATGCATC

3301 AAAGTATGCT TACACATTTA TCGACTGGGT GATTACAGCA GCTGCGAAAA AGAGACGAAA ATTAACTAAG GATAATTCTT GGCCAGAAAA CTTGTTATTA

3401 AACGTTAACG TTAAAAGTCT TGCATATATT TTAAGGATGA ATCGGTACAT CTGTACAAGG AACTGGAAAA AAATCGAGTT AGCTATCGAT AAATGTATAG

3501 AAATCGCCAT TAAGCTTGGC TGGTTATCTA GAAGAAAACG CATTGAATTT CTGGATTCTT CTAAACTCTC TAAAAAAGAA ATTCTATATC TAAATAAAGA

3601 GCGCTTTGAA GAAATAACTA AGAAATCTAA AGAACAAATG GAACAATTAG AACAAGAATC TATTAATTAA TAGCAAGCTT GAAACTAAAA ACCAGGGGGG

3701 ATCCTCCACG TTACTATGAT AAGTTACATT CTCGGTTTGA TCCGGAAGAG ATGGACGAGG TCAAACAAAA ACGTATAGAG AAAGTTATGG CTTTGCCTGA

3801 GCTAACTCAG GATAAGGCTG AGGTGAAGCA ATATATTTTC AATGACCGTA CGAAGAGACT CTTTAGAGAC TATGAGGAGG AGAGTTACTA AACTTTTTTA

3901 AAAAATAGGA GCTTTTTTCA ATGAAAGCTT GGGATCTCTA TAATCTCGCG CAACCTATTT TCCCCTCGAA CACTTTTTAA GCCGTAGATA AACAGGCTGG

4001 GACACTTCAC ATGAGCGAAA AATACATCGT CACCTGGGAC ATGTTGCAGA TCCATGCACG TAAACTCGCA AGCCGACTGA TGCCTTCTGA ACAATGGAAA

4101 GGCATTATTG CCGTAAGCCG TGGCGGTCTG GTACCGGTGG GTGAAGACCA GAAACAGCAC CTCGAACTGA GCCGCGATAT TGCCCAGCGT TTCAACGCGC

4201 TGTATGGCGA GATCGATCCC GTCGTTTTAC AACGTCGTGA CTGGGAAAAC CCTGGCGTTA CCCAACTTAA TCGCCTTGCA GCACATCCCC CTTTCGCCAG

4301 CTGGCGTAAT AGCGAAGAGG CCCGCACCGA TCGCCCTTCC CAACAGTTGC GCAGCCTGAA TGGCGAATGG CGCTTTGCCT GGTTTCCGGC ACCAGAAGCG

4401 GTGCCGGAAA GCTGGCTGGA GTGCGATCTT CCTGAGGCCG ATACTGTCGT CGTCCCCTCA AACTGGCAGA TGCACGGTTA CGATGCGCCC ATCTACACCA

4501 ACGTGACCTA TCCCATTACG GTCAATCCGC CGTTTGTTCC CACGGAGAAT CCGACGGGTT GTTACTCGCT CACATTTAAT GTTGATGAAA GCTGGCTACA

4601 GGAAGGCCAG ACGCGAATTA TTTTTGATGG CGTTAACTCG GCGTTTCATC TGTGGTGCAA CGGGCGCTGG GTCGGTTACG GCCAGGACAG TCGTTTGCCG

4701 TCTGAATTTG ACCTGAGCGC ATTTTTACGC GCCGGAGAAA ACCGCCTCGC GGTGATGGTG CTGCGCTGGA GTGACGGCAG TTATCTGGAA GATCAGGATA

4801 TGTGGCGGAT GAGCGGCATT TTCCGTGACG TCTCGTTGCT GCATAAACCG ACTACACAAA TCAGCGATTT CCATGTTGCC ACTCGCTTTA ATGATGATTT

4901 CAGCCGCGCT GTACTGGAGG CTGAAGTTCA GATGTGCGGC GAGTTGCGTG ACTACCTACG GGTAACAGTT TCTTTATGGC AGGGTGAAAC GCAGGTCGCC

5001 AGCGGCACCG CGCCTTTCGG CGGTGAAATT ATCGATGAGC GTGGTGGTTA TGCCGATCGC GTCACACTAC GTCTGAACGT CGAAAACCCG AAACTGTGGA

5101 GCGCCGAAAT CCCGAATCTC TATCGTGCGG TGGTTGAACT GCACACCGCC GACGGCACGC TGATTGAAGC AGAAGCCTGC GATGTCGGTT TCCGCGAGGT

5201 GCGGATTGAA AATGGTCTGC TGCTGCTGAA CGGCAAGCCG TTGCTGATTC GAGGCGTTAA CCGTCACGAG CATCATCCTC TGCATGGTCA GGTCATGGAT

5301 GAGCAGACGA TGGTGCAGGA TATCCTGCTG ATGAAGCAGA ACAACTTTAA CGCCGTGCGC TGTTCGCATT ATCCGAACCA TCCGCTGTGG TACACGCTGT

5401 GCGACCGCTA CGGCCTGTAT GTGGTGGATG AAGCCAATAT TGAAACCCAC GGCATGGTGC CAATGAATCG TCTGACCGAT GATCCGCGCT GGCTACCGGC

5501 GATGAGCGAA CGCGTAACGC GAATGGTGCA GCGCGATCGT AATCACCCGA GTGTGATCAT CTGGTCGCTG GGGAATGAAT CAGGCCACGG CGCTAATCAC

5601 GACGCGCTGT ATCGCTGGAT CAAATCTGTC GATCCTTCCC GCCCGGTGCA GTATGAAGGC GGCGGAGCCG ACACCACGGC CACCGATATT ATTTGCCCGA

5701 TGTACGCGCG CGTGGATGAA GACCAGCCCT TCCCGGCTGT GCCGAAATGG TCCATCAAAA AATGGCTTTC GCTACCTGGA GAGACGCGCC CGCTGATCCT

5801 TTGCGAATAC GCCCACGCGA TGGGTAACAG TCTTGGCGGT TTCGCTAAAT ACTGGCAGGC GTTTCGTCAG TATCCCCGTT TACAGGGCGG CTTCGTCTGG

5901 GACTGGGTGG ATCAGTCGCT GATTAAATAT GATGAAAACG GCAACCCGTG GTCGGCTTAC GGCGGTGATT TTGGCGATAC GCCGAACGAT CGCCAGTTCT

6001 GTATGAACGG TCTGGTCTTT GCCGACCGCA CGCCGCATCC AGCGCTGACG GAAGCAAAAC ACCAGCAGCA GTTTTTCCAG TTCCGTTTAT CCGGGCAAAC

6101 CATCGAAGTG ACCAGCGAAT ACCTGTTCCG TCATAGCGAT AACGAGCTCC TGCACTGGAT GGTGGCGCTG GATGGTAAGC CGCTGGCAAG CGGTGAAGTG

6201 CCTCTGGATG TCGCTCCACA AGGTAAACAG TTGATTGAAC TGCCTGAACT ACCGCAGCCG GAGAGCGCCG GGCAACTCTG GCTCACAGTA CGCGTAGTGC

6301 AACCGAACGC GACCGCATGG TCAGAAGCCG GGCACATCAG CGCCTGGCAG CAGTGGCGTC TGGCGGAAAA CCTCAGTGTG ACGCTCCCCG CCGCGTCCCA

6401 CGCCATCCCG CATCTGACCA CCAGCGAAAT GGATTTTTGC ATCGAGCTGG GTAATAAGCG TTGGCAATTT AACCGCCAGT CAGGCTTTCT TTCACAGATG

6501 TGGATTGGCG ATAAAAAACA ACTGCTGACG CCGCTGCGCG ATCAGTTCAC CCGTGCACCG CTGGATAACG ACATTGGCGT AAGTGAAGCG ACCCGCATTG

6601 ACCCTAACGC CTGGGTCGAA CGCTGGAAGG CGGCGGGCCA TTACCAGGCC GAAGCAGCGT TGTTGCAGTG CACGGCAGAT ACACTTGCTG ATGCGGTGCT

6701 GATTACGACC GCTCACGCGT GGCAGCATCA GGGGAAAACC TTATTTATCA GCCGGAAAAC CTACCGGATT GATGGTAGTG GTCAAATGGC GATTACCGTT

6801 GATGTTGAAG TGGCGAGCGA TACACCGCAT CCGGCGCGGA TTGGCCTGAA CTGCCAGCTG GCGCAGGTAG CAGAGCGGGT AAACTGGCTC GGATTAGGGC

6901 CGCAAGAAAA CTATCCCGAC CGCCTTACTG CCGCCTGTTT TGACCGCTGG GATCTGCCAT TGTCAGACAT GTATACCCCG TACGTCTTCC CGAGCGAAAA

7001 CGGTCTGCGC TGCGGGACGC GCGAATTGAA TTATGGCCCA CACCAGTGGC GCGGCGACTT CCAGTTCAAC ATCAGCCGCT ACAGTCAACA GCAACTGATG

7101 GAAACCAGCC ATCGCCATCT GCTGCACGCG GAAGAAGGCA CATGGCTGAA TATCGACGGT TTCCATATGG GGATTGGTGG CGACGACTCC TGGAGCCCGT

7201 CAGTATCGGC GGAATTCCAG CTGAGCGCCG GTCGCTACCA TTACCAGTTG GTCTGGTGTC AAAAATAATA ATAACCGGGC AGGCCATGTC TGCCCGTATT

7301 TCGCGTAAGG AAATCCATTA TGTACTATTT AAAAAACACA AACTTTTGGA TGTTCGGTTT ATTCTTTTTC TTTTACTTTT TTATCATGGG AGCCTACTTC

7401 CCGTTTTTCC CGATTTGGCT ACATGACATC AACCATATCA GCAAAAGTGA TACGGGTATT ATTTTTGCCG CTATTTCTCT GTTCTCGCTA TTATTCCAAC

7501 CGCTGTTTGG TCTGCTTTCT GACAAACTCG GAACTTGTTT ATTGCAGCTT ACCTGATTCA TCAGGCATTC CTAATTTATG TAGTCTAAGA ACCAGTATTA

7601 CTAATACAGG ATTGACTCCG ACAACGTATT CATTACGTGT AGGCGGTTTA GAAAGCGGTG TGGTATGGGT TAATGCCCTT TCTAATGGCA ATGATATTTT

7701 AGGAATAACA AATACTTCTA ATGTATCTTT TTTAGAGGTA ATACCTCAAA CAAACGCTTA AACAATTTTT ATTGGATTTT TCTTATAGGT TTTATATTTA

7801 GAGAAAACAG TTCGAATTAC GGGGTTTGTT ATGCAAAATA AAAGAAAAGT GAGGGACGAT TTTATTAAAA TTGTTAAAGA TGTGAAAAAA GATTTCCCCG

7901 AATTAGACCT AAAAATACGA GTAAACAAGG AAAAAGTAAC TTTCTTAAAT TCTCCCTTAG AACTCTACCA TAAAAGTGTC TCACTAATTC TAGGACTGCT

8001 TCAACAAATA GAAAACTCTT TAGGATTATT CCCAGACTCT CCTGTTCTTG AAAAATTAGA GGATAACAGT TTAAAGCTAA AAAAGGCTTT GATTATGCTT

8101 ATCTTGTCTA GAAAAGACAT GTTTTCCAAG GCTGAATAGA CAACTTACTC TAACGTTGGA GTTGATTTGC ACACCTTAGT TTTTTGCTCT TTTAAGGGAG

8201 GAACTGGAAA AACAACACTT TCTCTAAACG TGGGATGCAA CTTGGCCCAA TTTTTAGGGA AAAAAGTGTT ACTTGCTGAC CTAGACCCGC AATCCAATTT

8301 ATCTTCTGGA TTGGGGGCTA GTGTCAGAAG TGACCAAAAA GGCTTGCACG ACATAGTATA CACATCAAAC GATTTAAAAT CAATCATTTG CGAAACAAAA

8401 AAAGATAGTG TGGACCTAAT TCCTGCATCA TTTTCATCCG AACAGTTTAG AGAATTGGAT ATTCATAGAG GACCTAGTAA CAACTTAAAG TTATTTCTGA

8501 ATGAGTACTG CGCTCCTTTT TATGACATCT GCATAATAGA CACTCCACCT AGCCTAGGAG GGTTAACGAA AGAAGCTTTT GTTGCAGGAG ACAAATTAAT

8601 TGCTTGTTTA ACTCCAGAAC CTTTTTCTAT TCTAGGGTTA CAAAAGATAC GTGAATTCTT AAGTTCGGTC GGAAAACCTG AAGAAGAACA CATTCTTGGA

8701 ATAGCTTTGT CTTTTTGGGA TGATCGTAAC TCGACTAACC AAATGTATAT AGACATTATC GAGTCTATTT ACAAAAACAA GCTTTTTTCA ACAAAAATTC

8801 GTCGAGATAT TTCTCTCAGC CGTTCTCTTC TTAAAGAAGA TTCTGTAGCT AATGTCTATC CAAATTCTAG GGCCGCAGAA GATATTCTGA AGTTAACGCA

8901 TGAAATAGCA AATATTTTGC ATATCGAATA TGAACGAGAT TACTCTCAGA GGACAACGTG AACAAACTAA AAAAAGAAGC GGATGTCTTT TTTAAAAAAA

9001 ATCAAACTGC CGCTTCTCTA GATTTTAAGA AGACGCTTCC CTCCATTGAA CTATTCTCAG CAACTTTGAA TTCTGAGGAA AGTCAGAGTT TGGATCGATT

9101 ATTTTTATCA GAGTCCCAAA ACTATTCGGA TGAAGAATTT TATCAAGAAG ACATCCTAGC GGTAAAACTG CTTACTGGTC AGATAAAATC CATACAGAAG

9201 CAACACGTAC TTCTTTTAGG AGAAAAAATC TATAATGCTA GAAAAATCCT GAGTAAGGAT CACTTCTCCT CAACAACTTT TTCATCTTGG ATAGAGTTAG

9301 TTTTTAGAAC TAAGTCTTCT GCTTACAATG CTCTTGCATA TTACGAGCTT TTTATAAACC TCCCCAACCA AACTCTACAA AAAGAGTTTC AATCGATCCC

9401 CTATAAATCC GCATATATTT TGGCCGCTAG AAAAGGCGAT TTAAAAACCA AGGTCGATGT GATAGGGAAA GTATGTGGAA TGTCGAACTC ATCGGCGATA

9501 AGGGTGTTGG ATCAATTTCT TCCTTCATCT AGAAACAAAG ACGTTAGAGA AACGATAGAT AAGTCTGATT CAGAGAAGAA TCGCCAATTA TCTGATTTCT

9601 TAATAGAGAT ACTTCGCATC ATGTGTTCCG GAGTTTCTTT GTCCTCCTAT AACGAAAATC TTCTACAACA GCTTTTTGAA CTTTTTAAGC AAAAGAGCTG

9701 ATCCTCCGTC AGCTCATATA TATATATCTA TTATATATAT ATATTTAGGG ATTTGATTTC ACGAGAGAGA TTTGCAACTC TTGGTGGTAG ACTTTGCAAC

9801 TCTTGGTGGT AGACTTTGCA ACTCTTGGTG GTAGACTTTG CAACTCTTGG TGGTAGACTT GGTCATAATG GACTTTTGTT AAAAAATTTC TTAAAATCTT

9901 AGAGCTCCGA TTTTGAATAG CTTTGGTTAA GAAAATGGGC TCGATGGCTT TCCATAAAAG TAGATTGTTT TTAACTTTTG GGGACGCGTC GGAAATTTGG

10001 TTATCTACTT TATCTTATCT AACTAGAAAA AATTATGCGT CTGGGATTAA CTTTCTTGTT TCTTTAGAGA TTCTGGATTT ATCGGAAACC TTGATAAAGG

10101 CTATTTCTCT TGACCACAGC GAATCTTTGT TTAAAATCAA GTCTCTAGAT GTTTTTAATG GAAAAGTTGT TTCAGAGGCA TCTAAACAGG CTAGAGCGGC

10201 ATGCTACATA TCTTTCACAA AGTTTTTGTA TAGATTGACC AAGGGATATA TTAAACCCGC TATTCCATTG AAAGATTTTG GAAACACTAC ATTTTTTAAA

10301 ATCCGAGACA AAATCAAAAC AGAATCGATT TCTAAGCAGG AATGGACAGT TTTTTTTGAA GCGCTCCGGA TAGTGAATTA TAGAGACTAT TTAATCGGTA

10401 AATTGATTGT ACAAGGGATC CCCGGGTACC GAGCTCGAAT TCATCGATGA TATCAGATCT GGTTCTATAG TGTCACCTAA ATCGTATGTG TATGATACAT

10501 AAGGTTATGT ATTAATTGTA GCCGCGTTCT AACGACAATA TGTCCATATG GTGCACTCTC AGTACAATCT GCTCTGATGC CGCATAGTTA AGCCAGCCCC

10601 GACACCCGCC AACACCCGCT GACGCGCCCT GACGGGCTTG TCTGCTCCCG GCATCCGCTT ACAGACAAGC TGTGACCGTC TCCGGGAGCT GCATGTGTCA

10701 GAGGTTTTCA CCGTCATCAC CGAAACGCGC GAGACGAAAG GGCCTCGTGA TACGCCTATT TTTATAGGTT AATGTCATGA TAATAATGGT TTCTTAGACG

10801 TCAGGTGGCA CTTTTCGGGG AAATGTGCGC GGAACCCCTA TTTGTTTATT TTTCTAAATA CATTCAAATA TGTATCCGCT CATGAGACAA TAACCCTGAT

10901 AAATGCTTCA ATAATATTGA AAAAGGAAGA GTATGAGTAT TCAACATTTC CGTGTCGCCC TTATTCCCTT TTTTGCGGCA TTTTGCCTTC CTGTTTTTGC

11001 TCACCCAGAA ACGCTGGTGA AAGTAAAAGA TGCTGAAGAT CAGTTGGGTG CACGAGTGGG TTACATCGAA CTGGATCTCA ACAGCGGTAA GATCCTTGAG

11101 AGTTTTCGCC CCGAAGAACG TTTTCCAATG ATGAGCACTT TTAAAGTTCT GCTATGTGGC GCGGTATTAT CCCGTATTGA CGCCGGGCAA GAGCAACTCG

11201 GTCGCCGCAT ACACTATTCT CAGAATGACT TGGTTGAGTA CTCACCAGTC ACAGAAAAGC ATCTTACGGA TGGCATGACA GTAAGAGAAT TATGCAGTGC

11301 TGCCATAACC ATGAGTGATA ACACTGCGGC CAACTTACTT CTGACAACGA TCGGAGGACC GAAGGAGCTA ACCGCTTTTT TGCACAACAT GGGGGATCAT

11401 GTAACTCGCC TTGATCGTTG GGAACCGGAG CTGAATGAAG CCATACCAAA CGACGAGCGT GACACCACGA TGCCTGTAGC AATGGCAACA ACGTTGCGCA

11501 AACTATTAAC TGGCGAACTA CTTACTCTAG CTTCCCGGCA ACAATTAATA GACTGGATGG AGGCGGATAA AGTTGCAGGA CCACTTCTGC GCTCGGCCCT

11601 TCCGGCTGGC TGGTTTATTG CTGATAAATC TGGAGCCGGT GAGCGTGGGT CTCGCGGTAT CATTGCAGCA CTGGGGCCAG ATGGTAAGCC CTCCCGTATC

11701 GTAGTTATCT ACACGACGGG GAGTCAGGCA ACTATGGATG AACGAAATAG ACAGATCGCT GAGATAGGTG CCTCACTGAT TAAGCATTGG TAACTGTCAG

11801 ACCAAGTTTA CTCATATATA CTTTAGATTG ATTTAAAACT TCATTTTTAA TTTAAAAGGA TCTAGGTGAA GATCCTTTTT GATAATCTCA TGACCAAAAT

11901 CCCTTAACGT GAGTTTTCGT TCCACTGAGC GTCAGACCCC GTAGAAAAGA TCAAAGGATC TTCTTGAGAT CCTTTTTTTC TGCGCGTAAT CTGCTGCTTG

12001 CAAACAAAAA AACCACCGCT ACCAGCGGTG GTTTGTTTGC CGGATCAAGA GCTACCAACT CTTTTTCCGA AGGTAACTGG CTTCAGCAGA GCGCAGATAC

12101 CAAATACTGT CCTTCTAGTG TAGCCGTAGT TAGGCCACCA CTTCAAGAAC TCTGTAGCAC CGCCTACATA CCTCGCTCTG CTAATCCTGT TACCAGTGGC

12201 TGCTGCCAGT GGCGATAAGT CGTGTCTTAC CGGGTTGGAC TCAAGACGAT AGTTACCGGA TAAGGCGCAG CGGTCGGGCT GAACGGGGGG TTCGTGCACA

12301 CAGCCCAGCT TGGAGCGAAC GACCTACACC GAACTGAGAT ACCTACAGCG TGAGCATTGA GAAAGCGCCA CGCTTCCCGA AGGGAGAAAG GCGGACAGGT

12401 ATCCGGTAAG CGGCAGGGTC GGAACAGGAG AGCGCACGAG GGAGCTTCCA GGGGGAAACG CCTGGTATCT TTATAGTCCT GTCGGGTTTC GCCACCTCTG

12501 ACTTGAGCGT CGATTTTTGT GATGCTCGTC AGGGGGGCGG AGCCTATGGA AAAACGCCAG CAACGCGGCC TTTTTACGGT TCCTGGCCTT TTGCTGGCCT

12601 TTTGCTCACA TGTTCTTTCC TGCGTTATCC CCTGATTCTG TGGATAACCG TATTACCGCC TTTGAGTGAG CTGATACCGC TCGCCGCAGC CGAACGACCG

12701 AGCGCAGCGA GTCAGTGAGC GAGGAAGCGG AAGAGCGCCC AATACGCAAA CCGCCTCTCC CCGCGCGTTG GCCGATTCAT TAATGCAGGT TAACCTGGCT

12801 TATCGAAATT AATACGACTC ACTATAGGGA GACCGGCCTC GAGCAGCTGA AGCTTGCATG CCTGCAGATG CCCGACGGTC TTTATAGCGG ATTAACAAAA

12901 ATCAGGACAA GGCGGCGAAG CCGAAGACAG TACAAATAGC ACGGAACCGA TTCACTTGGT GCTTCAGCAC CTTAGAGAAT CGTTCTCTTT GAGCTAAGGC

13001 GAGGCAACGC CGTACTTGTT TTTGTTAATC CACTATAAAG TGCCGCGTGT GTTTTTTTAT GGCGTTTTAA AAAGCCGAGA CTGCATCCGG GCAGCAGCGC

13101 ATCGGCCCGC ACGAGGTCTG CGCTTGAATT GTGTTGTAGA AACACAACGT TTTTTGAAAA AATAAGCTAT TGTTTTATAT CAAAATATAA TCATTTTTAA

13201 AATAAAGGTT GCGGCATTTA TCAGATATTT GTTCTGAAAA ATGGTTTTTT GCGGGGGGGG GGGTATAATT GAAGACGTAT CGGGTGTTTG CCCGATGTTT

13301 TTAGGTTTTT ATCAAATTTA CAAAAGGAAG CCGATATGGT GGATCCCCGG GTACCAATGA GTAAAGGAGA AGCACTTTTC ACTGGAGTTG TCCCAATTCT

13401 TGTTGAATTA GATGGTGATG TTAATGGGCA CAAATTTTCT GTCAGTGGAG AGGGTGAAGG TGATGCAACA TACGGAAAAC TTACCCTTAA ATTTATTTGC

13501 ACTACTGGAA AACTACCTGT TCCATGGCCA ACACTTGTCA CTACTCTTAC GTATGGTGTT CAATGCTTTT CAAGATACCC AGATCATATG AAACGGCATG

13601 ACTTTTTCAA GAGTGCCATG CCCGAAGGTT ATGTACAGGA AAGAACTATA TTTTTCAAAG ATGACGGGAA CTACAAGACA CGTGCTGAAG TCAAGTTTGA

13701 AGGTGATACC CTTGTTAATA GAATCGAGTT AAAAGGTATT GATTTTAAAG AAGATGGAAA CATTCTTGGA CACAAATTGG AATACAACTA TAACTCACAC

13801 AATGTATACA TCATGGCAGA CAAACAAAAG AATGGAATCA AAGTTAACTT CAAAATTAGA CACAACATTG AAGATGGAAG CGTTCAACTA GCAGACCATT

13901 ATCAACAAAA TACTCCAATT GGCGATGGCC CTGTCCTTTT ACCAGACAAC CATTACCTGT CCACACAATC TGCCCTTTCG AAAGATCCCA ACGAAAAGAG

14001 AGACCACATG GTCCTTCTTG AGTTTGTAAC AGCTGCTGGG ATTACACATG GCATGGATGA ACTATACAAG TCCGGACTCA GATCTATGGA GAAAAAAATC

14101 ACTGGATATA CCACCGTTGA TATATCCCAA TGGCATCGCA AAGAACATTT TGAGGCATTT CAGTCAGTTG CTCAATGTAC CTATAACCAG ACCGTTCAGC

14201 TGGATATTAC GGCCTTTTTA AAGACCGTAA AGAAAAATAA GCACAAGTTT TATCCGGCCT TTATTCACAT TCTTGCCCGC CTGATGAATG CTCATCCGGA

14301 ATTCCGTATG GCAATGAAAG ACGGTGAGCT GGTGATATGG GATAGTGTTC ACCCTTGTTA CACCGTTTTC CATGAGCAAA CTGAAACGTT TTCATCGCTC

14401 TGGAGTGAAT ACCACGACGA TTTCCGGCAG TTTCTACACA TATATTCGCA AGATGTGGCG TGTTACGGTG AAAACCTGGC CTATTTCCCT AAAGGGTTTA

14501 TTGAGAATAT GTTTTTCGTC TCAGCCAATC CCTGGGTGAG TTTCACCAGT TTTGATTTAA ACGTGGCCAA TATGGACAAC TTCTTCGCCC CCGTTTTCAC

14601 CATGGGCAAA TATTATACGC AAGGCGACAA GGTGCTGATG CCGCTGGCGA TTCAGGTTCA TCATGCCGTT TGTGATGGCT TCCATGTCGG CAGAATGCTT

14701 AATGAATTAC AACAGTACTG CGATGAGTGG CAGGGCGGGG CGTAAAGATC TCGAGCTCGA TATCTAGATT AATG
