# Supplementary figures and images for: Genetic Transformation of a Clinical (Genital Tract), Plasmid-Free Isolate of Chlamydia trachomatis: Engineering the Plasmid as a Cloning Vector
Source: PLoS One. 2013 Mar 18;8(3):e59195. doi: 10.1371/journal.pone.0059195 (PMC3601068; doi:10.1371/journal.pone.0059195)

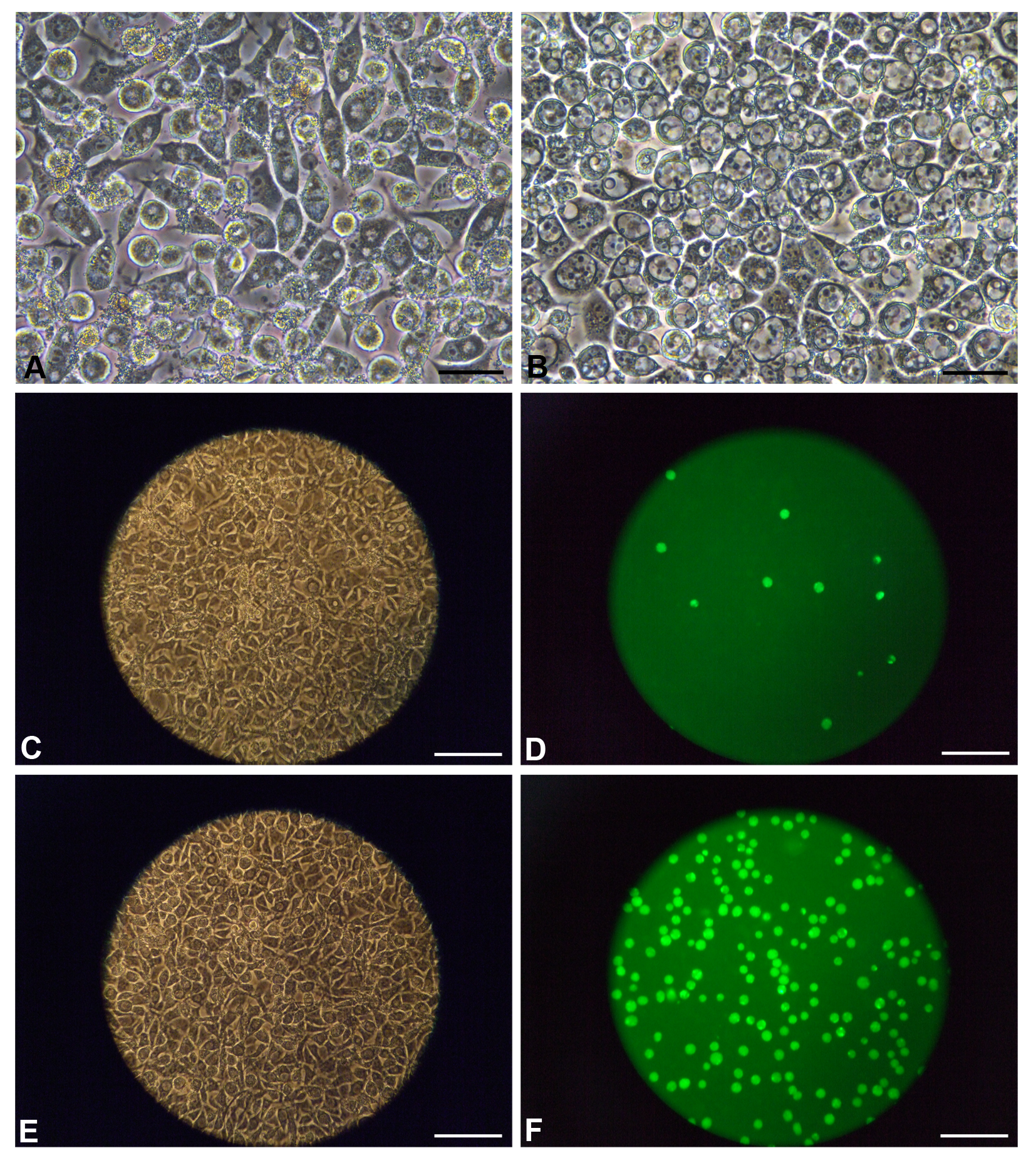

Supplement: Figure S3 — Microscopic images showing the early stages of recovery for transformants of C. trachomatis SWFP- with plasmid pGFP::SW2. The transformation mix (C. trachomatis SWFP-, plasmid DNA and McCoy cells in CaCl2/Tris buffer) was seeded onto 6-well plate in penicillin-free medium for two days before harvest T0 (A). Recovery of transformants was performed by 2–3 rounds of passages under penicillin selection (10 units/ml) in McCoy cells in T25 flasks (B–F). After two days in Passage 1, all inclusions appeared to be ‘abnormal’ (B). Nevertheless, after two days in Passage 2, ‘normal’ inclusions begin to emerge (C&D, D is the same field as C under blue light). More transformants were grown in Passage 3 (E&F, F is the same filed as E under blue light). Scale bars: 40 µm in images A&B and 100 µm in C–F. (TIF) [file pone.0059195.s003.tif]

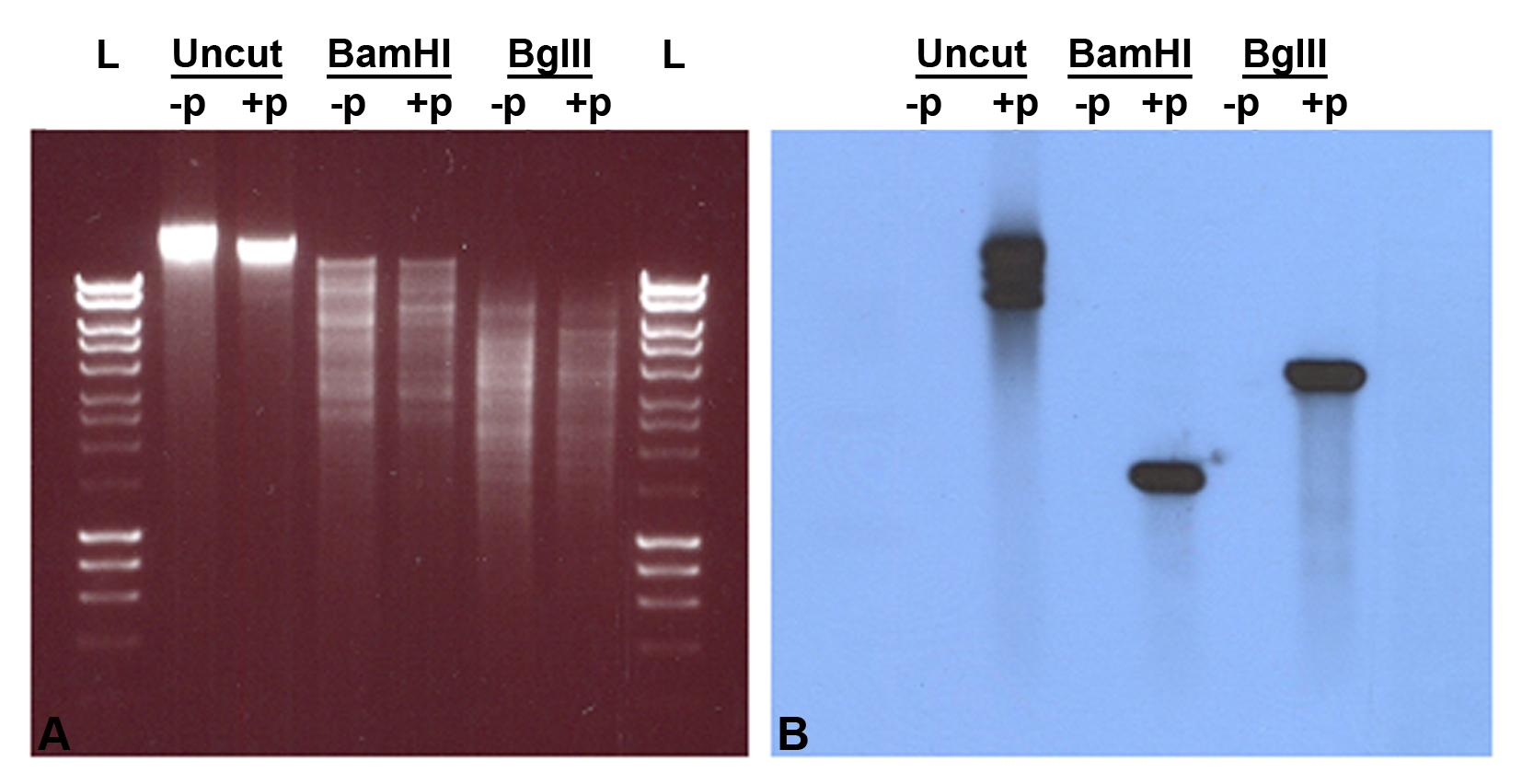

Supplement: Figure S4 — Southern blot of C. trachomatis SWFP- (−p) and C. trachomatis SWFP- transformed by plasmid pGFP::SW2 (+p) using a DIG-labelled GFP probe. Six chlamydial genomic DNA samples (∼0.5 µg DNA/lane) were loaded on 1% agarose gel in pairs together with HyperLadder I (5 µl) from BIOLINE (Cat No. BIO-33025). The agarose gel image was taken before DNA transfer (A). The DNA blot was hybridized with the GFP probe (B). The Bam HI digestion of pGFP::SW2 generated 3 fragments: 7169 bp, 2925 bp and 1445 bp (containing GFP probe sequence). The Bgl II digestion of pGFP::SW2 generated 4 fragments: 5555 bp, 3625 bp (containing the GFP probe sequence), 1693 bp and 666 bp. The Southern blot showed that the hybridization signals at expected positions in all SWFP−/pGFP::SW2 samples (uncut or digested); whilst no hybridization signal was detected in all SWFP- samples (uncut or digested) (B). (TIF) [file pone.0059195.s004.tif]

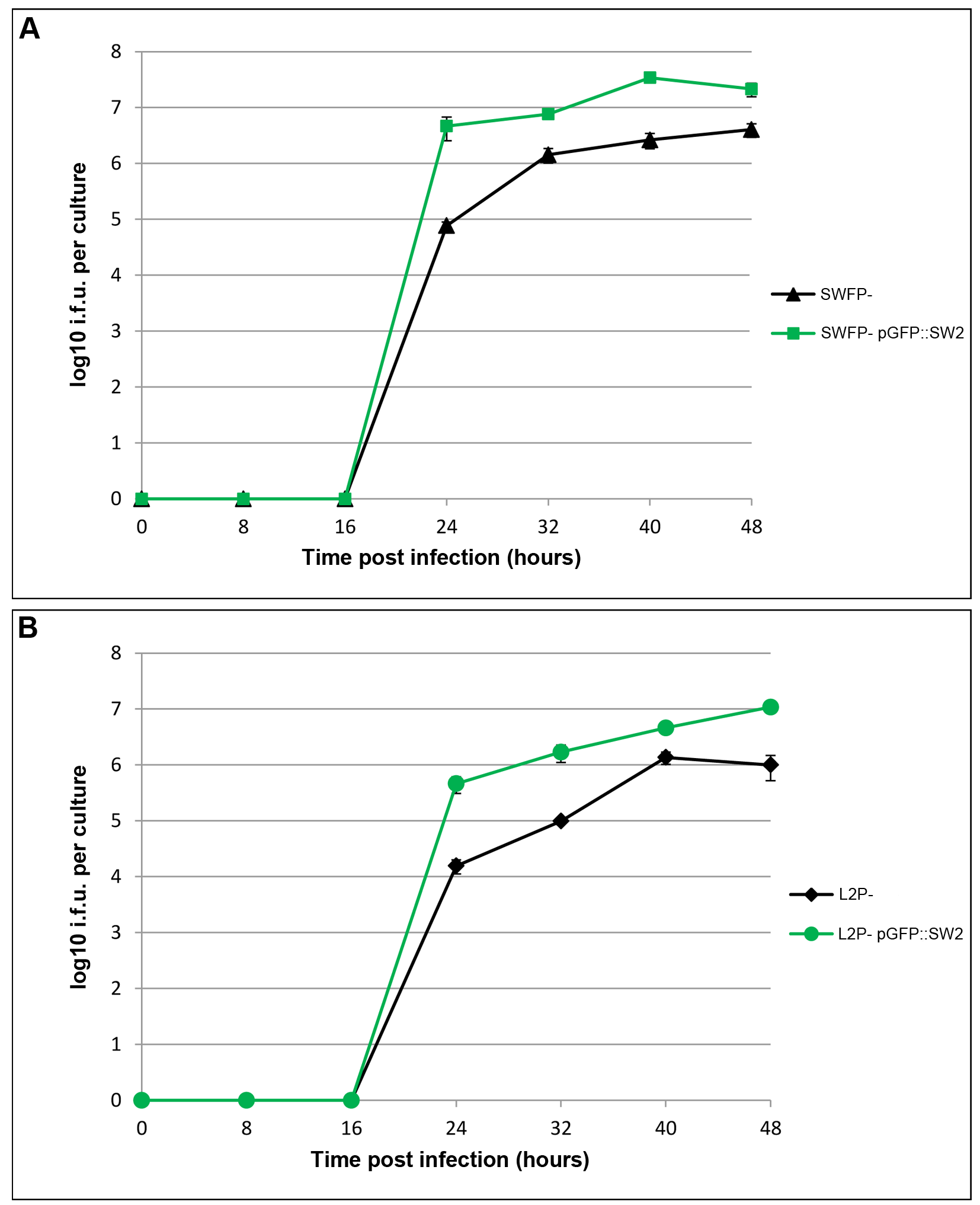

Supplement: Figure S5 — C. trachomatis SWFP- and C. trachomatis L2 (25667R) grow faster and give a higher yield when transformed by pGFP::SW2. (A) C. trachomatis SWFP- (black) and C. trachomatis SWFP- transformed with pGFP::SW2 (green) (B) C. trachomatis L2 (25667R) and C. trachomatis L2 (25667R) transformed with pGFP::SW2. McCoy cells in a 24 well tissue culture tray grown to confluence were infected with C. trachomatis at MOI = 1 and were cultured as described. The yield of C. trachomatis (IFU) per culture or single well is shown on the y – axis and time of sampling post infection is shown on the x –axis. The experiments were repeated in quadruplicate and standard error bars are shown for each sample point. (TIF) [file pone.0059195.s005.tif]

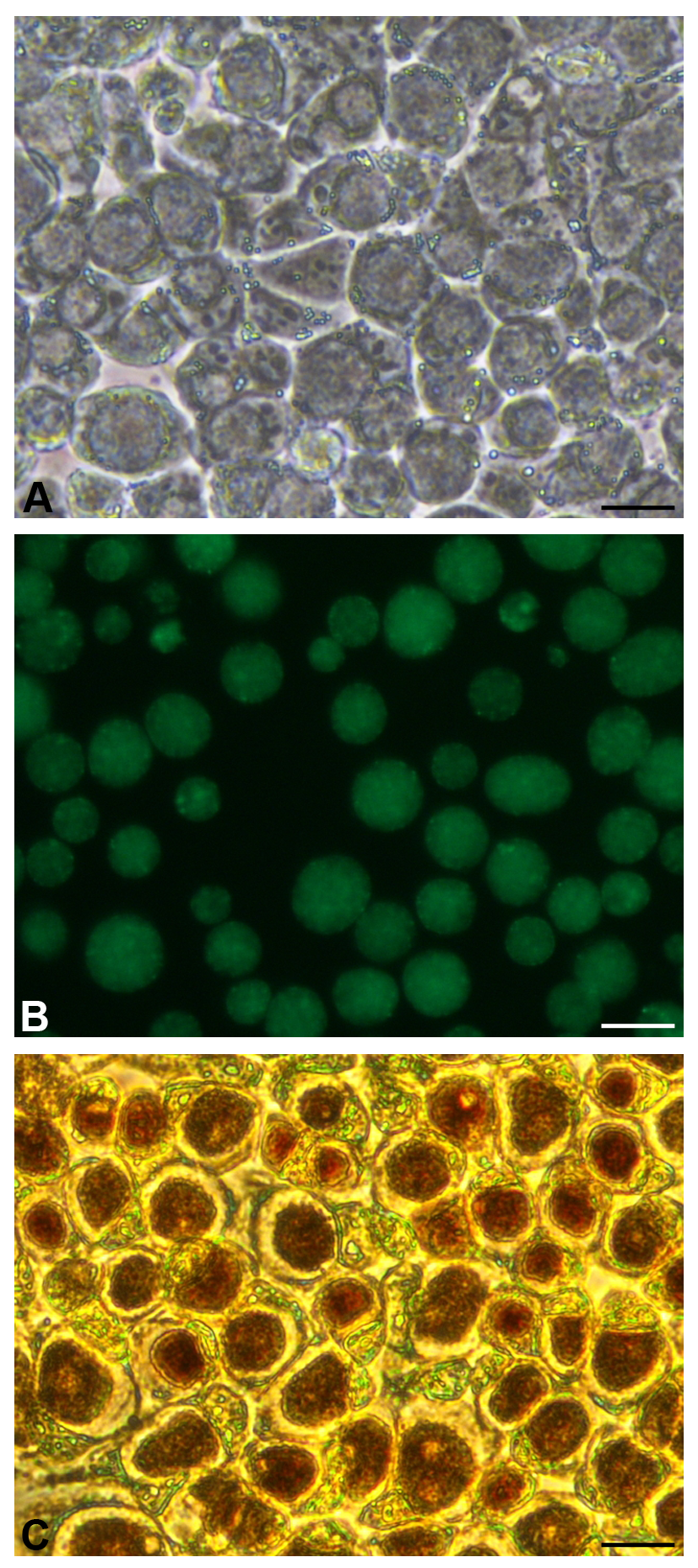

Supplement: Figure S6 — Properties of C. trachomatis SWFP- transformed with plasmid pCDS5KO (SWFP−/pCDS5KO). (A) Image of live McCoy cells infected with SWFP−/pCDS5KO under white light (phase contrast). (B) The same field as (A) under blue light. (C) Iodine stained McCoy cells (Methanol-fixed on coverslips) infected with SWFP−/pCDS5KO. The transformant SWFP−/pCDS5KO expressed the green fluorescent protein and was iodine-stain positive. Scale bar represents 20 µm. (TIF) [file pone.0059195.s006.tif]

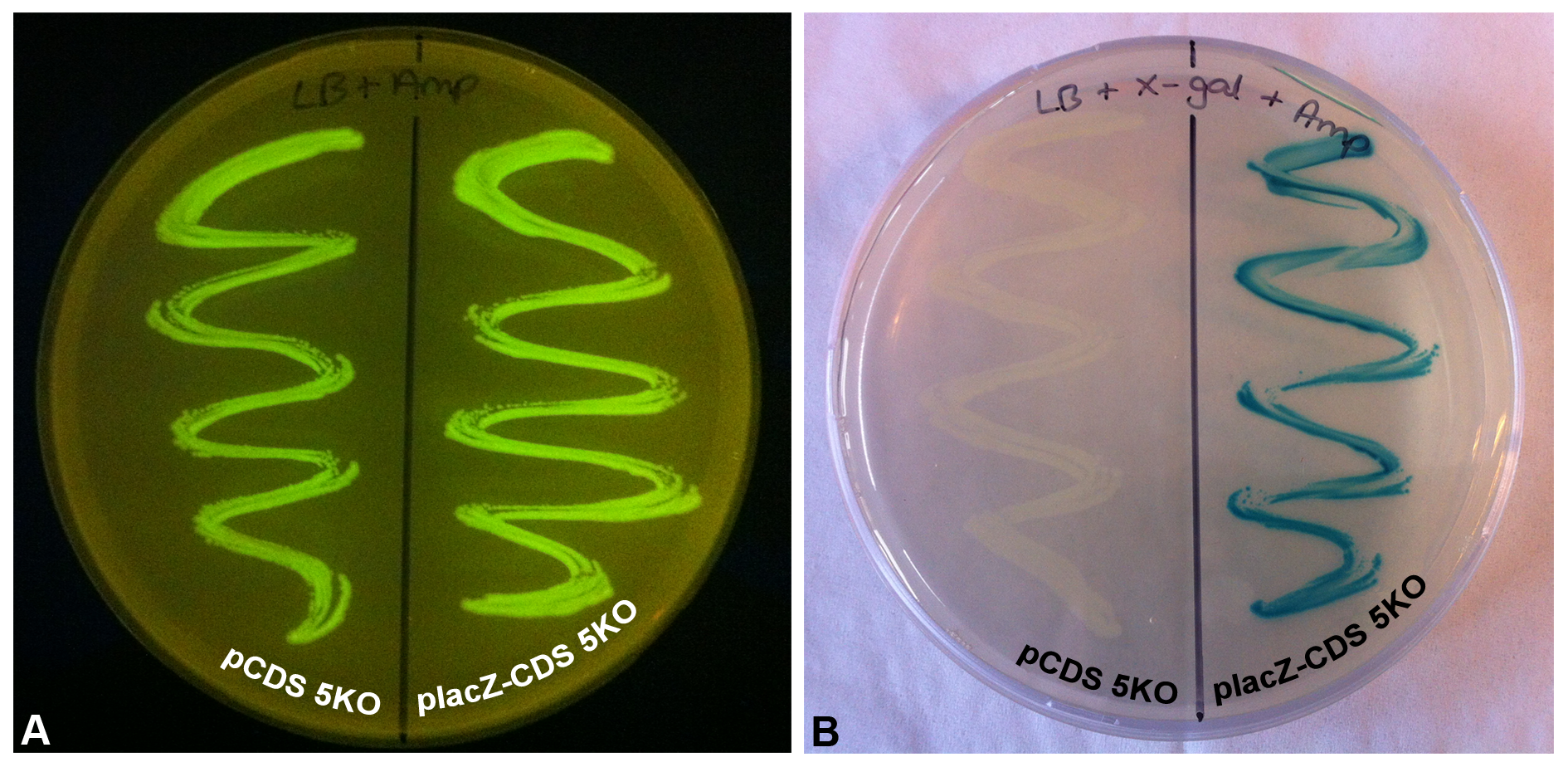

Supplement: Figure S7 — Phenotypic properties E. coli transformed with plasmids pCDS5KO and placZ-CDS5KO. E. coli strain MC1061 transformed with pCDS5KO and placZ-CDS5KO was grown on (A) an LB-amp agar plate, and (B) LB-Xgal-amp agar plate. Expression of the green fluorescent protein was visualised under blue light. (TIF) [file pone.0059195.s007.tif]

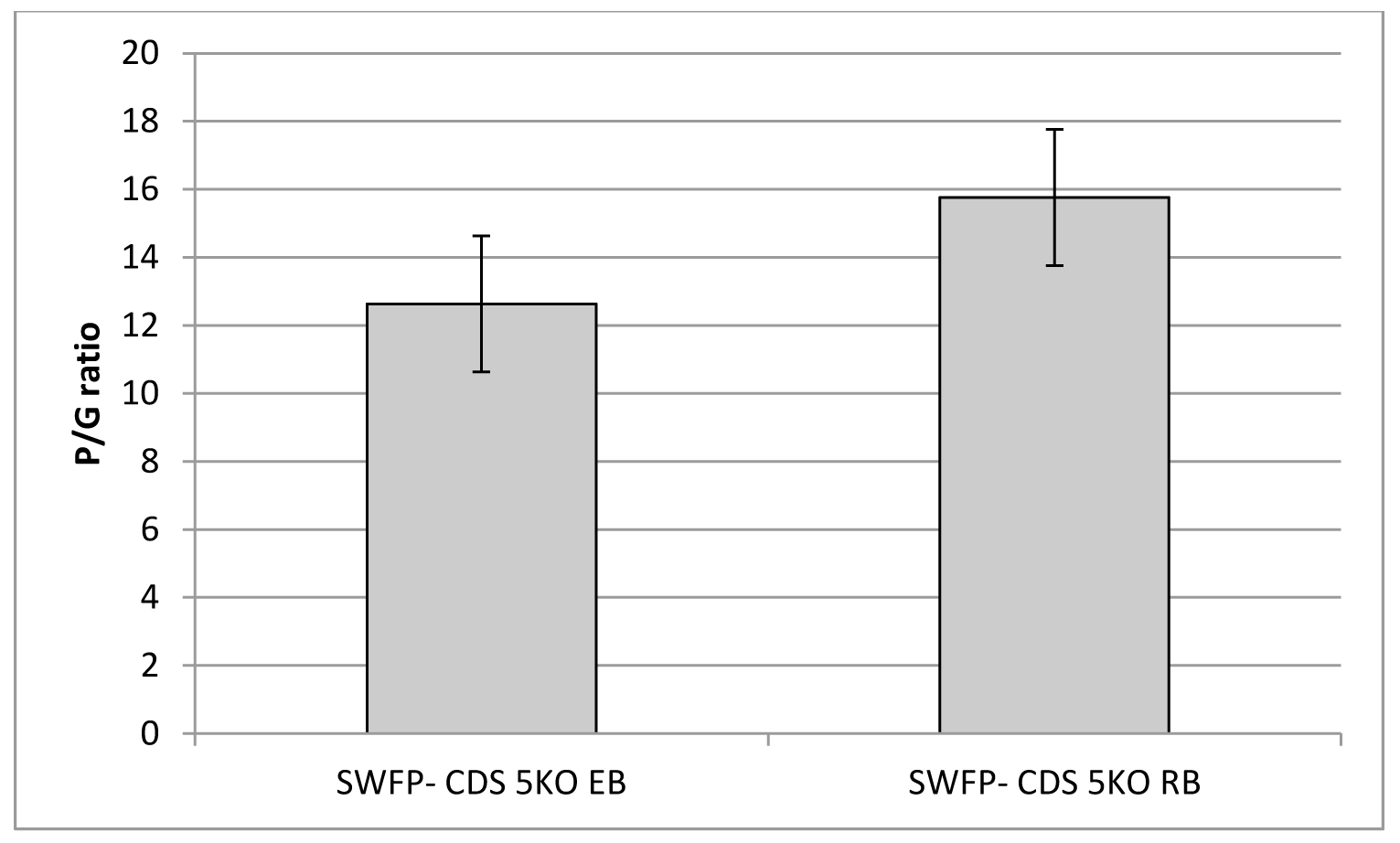

Supplement: Figure S8 — Plasmid copy number in C. trachomatis SWFP- transformed by pCDS5KO. Genomic DNA was extracted from gradient-purified EBs and RBs and analysed by qPCR to determine the plasmid/genome ratios (P/G). Standard error bars are shown. (TIF) [file pone.0059195.s008.tif]
